# Novel endogenous *N*-acyl amides activate TRPV1-4 receptors, BV-2 microglia, and are regulated in brain in an acute model of inflammation

**DOI:** 10.3389/fncel.2014.00195

**Published:** 2014-08-01

**Authors:** Siham Raboune, Jordyn M. Stuart, Emma Leishman, Sara M. Takacs, Brandon Rhodes, Arjun Basnet, Evan Jameyfield, Douglas McHugh, Theodore Widlanski, Heather B. Bradshaw

**Affiliations:** ^1^Department of Psychological and Brain Sciences, Indiana UniversityBloomington, IN, USA; ^2^Department of Chemistry, Indiana UniversityBloomington IN, USA

**Keywords:** lipid signaling, endocannabinoids, N-acyl amides, TRP receptors, microglia

## Abstract

A family of endogenous lipids, structurally analogous to the endogenous cannabinoid, *N*-arachidonoyl ethanolamine (Anandamide), and called *N*-acyl amides have emerged as a family of biologically active compounds at TRP receptors. *N*-acyl amides are constructed from an acyl group and an amine via an amide bond. This same structure can be modified by changing either the fatty acid or the amide to form potentially hundreds of lipids. More than 70 *N*-acyl amides have been identified in nature. We have ongoing studies aimed at isolating and characterizing additional members of the family of *N*-acyl amides in both central and peripheral tissues in mammalian systems. Here, using a unique in-house library of over 70 *N*-acyl amides we tested the following three hypotheses: (1) Additional *N*-acyl amides will have activity at TRPV1-4, (2) Acute peripheral injury will drive changes in CNS levels of *N*-acyl amides, and (3) *N*-acyl amides will regulate calcium in CNS-derived microglia. Through these studies, we have identified 20 novel *N*-acyl amides that collectively activate (stimulating or inhibiting) TRPV1-4. Using lipid extraction and HPLC coupled to tandem mass spectrometry we showed that levels of at least 10 of these *N*-acyl amides that activate TRPVs are regulated in brain after intraplantar carrageenan injection. We then screened the BV2 microglial cell line for activity with this *N*-acyl amide library and found overlap with TRPV receptor activity as well as additional activators of calcium mobilization from these lipids. Together these data provide new insight into the family of *N*-acyl amides and their roles as signaling molecules at ion channels, in microglia, and in the brain in the context of inflammation.

## Introduction

Transient receptor potential channels (TRPs) form a large family of ubiquitous ligand-gated non-selective cation channels that function as cellular sensors and in many cases regulate intracellular calcium. Identification of the endogenous ligands that activate the majority of TRP receptors is still under intense investigation with most of these channels still remaining “orphans” (Vriens et al., 2004; Nilius and Owsianik, 2011; Beech, 2012). Much work has shown that TRPs respond to a variety of external stimuli (e.g., plant-derived lipids), however, most TRP receptors are expressed in areas that would not come in direct contact with external stimuli (e.g., CNS tissue, internal organs, smooth muscle); therefore, the mechanism of direct activation by cellular messengers is still under much debate and research. In the few cases where endogenous ligands have been identified that drive direct activation of TRPs, the majority of these ligands are small lipid molecules, most of which share structural homology (Bradshaw et al., 2013).

Members of the TRPV family that consists of TRPV1-6, of which TRPV1-4 (also known as the thermoTRPs for their responses to heat) are the most targeted TRP receptors for drug discovery as they are major players in mechanisms of pain and inflammation (Di Marzo et al., 2002; Julius, 2013). TRPV1 (formerly VR1) receptors identification in dorsal root ganglia (DRG) in 1997 was shown to be a molecular integrator of different types of noxious stimuli (Caterina et al., 1997). TRPV1 receptors are mainly expressed in neuronal tissue such as Aδ and C fibers as well as DRG, trigeminal and nodose ganglia, and sensory neurons of the jugular ganglia as well as many brain areas (Tominaga et al., 1998; Caterina and Julius, 2001; Nagy et al., 2004; Starowicz et al., 2008; Cavanaugh et al., 2011a,b). Yet the complete picture of the role of TRPV activity in the CNS is still largely unknown and less still is known about brain TRP activity in the context of pain and inflammation in the brain. Unlocking the mysteries of TRP channel activation will have far-reaching effects on our understanding of cell signaling and pathophysiology in the CNS.

**Figure 1:**
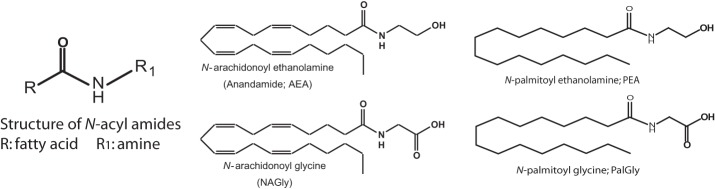
**Structure of *N*-acyl amides**. *N*-acyl amides studied here have a structure that consists of an acyl group (denoted as R) and an amide bond to an amine (denoted as R_1_) and this is depicted as a cartoon structure here (far left). 4 examples of *N*-acyl amide bioactive molecules are also depicted: *N*-arachidonoyl ethanolamide (Anandamide; AEA); *N*-arachidonoy glycine (NAGly); *N*-palmitoyl ethanolamide (PEA); and *N*-palmitoyl glycine (PalGly). These 4 N-acyl amide species illustrate that the molecules can differ by changing the acyl group (AEA and PEA) or the amide (AEA and NAGly).

Lipidomics, the field of study focused on identifying and characterizing the full complement of biological lipids, has identified a class of endogenous lipids, *N*-acyl amides, which are providing a novel avenue from which to studying cellular communication. *N*-arachidonoyl ethanolamine, (Anandamide—the first endogenous cannabinoid identified) is an archetype *N*-acyl amide that is constructed from an acyl group, arachidonic acid, and an amine, ethanolamine via an amide bond. This same structure can be modified by changing either the fatty acid or the amide to form potentially hundreds of lipids (Figure 1). More than 70 *N*-acyl amides have been identified in nature and our group has been a driving force behind this work (Bradshaw and Walker, 2005; Tan et al., 2006; Burstein et al., 2007; Hu et al., 2008; Rimmerman et al., 2008; Bradshaw et al., 2009; Hu et al., 2009; Lee et al., 2010; Mchugh et al., 2010; Smoum et al., 2010; Tortoriello et al., 2013).

Multiple members of the *N*-acyl amide class of lipids have been shown to activate a member of the TRPV family (Di Marzo et al., 2002; Julius, 2013). Anandamide was the first *N*-acyl amide shown to activate TRPV1 channels (Zygmunt et al., 1999). *N*-arachidonoyl dopamine (NADA) and *N*-oleoyl dopamine (OLDA), which are structurally similar to the exogenous TRPV1 agonist capsaicin as well as *N*-acyl amide structural analogs to AEA, are more potent endogenous ligand for TRPV1 receptors (Huang et al., 2002; Chu et al., 2003). Additional work showed that *N*-arachidonoyl taurine, which does not activate CB receptors, actives both TRPV1 and TRPV4 receptors (Saghatelian et al., 2006). Likewise, a broader range of N-acyl ethanolamides were shown to activate TRPV1 (Movahed et al., 2005), further demonstrating that non-eCB type *N*-acyl amides could also activate TRPs.

Here, we used a unique in-house library of over 70 *N*-acyl amides (for complete listed see Supplemental Table 1) we tested the following three hypotheses: (1) Additional *N*-acyl amides will have activity at TRPV1-4, (2) Acute peripheral injury will drive changes in CNS levels of *N*-acyl amides, and (3) *N*-acyl amides will regulate calcium in CNS-derived microglia. Through these studies we identified 20 novel *N*-acyl amide activators of these TRPVs collectively. We then performed targeted lipidomics using the *N*-acyl amide library on 6 brain regions to determine if acute inflammation by intraplantar paw injection of carrageenan would drive changes in these lipids. Here, we demonstrated that a wide range of lipids are regulated in the brain during acute inflammation and at least 10 of these newly identified TRPV activator lipids are regulated in the brain at 3 h post injection suggesting that this family of lipids plays important roles in CNS signaling. Finally, we screened BV2 microglial cells with the *N*-acyl amide library to determine if *N*-acyl amides play a role in microglial signaling. In these experiments we show that 6 novel *N*-acyl amide families of lipids active these microglial cells, 5 of which had members that were upregulated 3 h post-carrageenan injection in the brain. Data presented here provide evidence that the TRPV and *N*-acyl amide systems may work together to respond to rapid changes in the neurophysiological environment of acute pain.

## Materials and methods

### Chemicals

Fura-2 AM was purchased from Invitrogen (Carlsbad, CA). 5-Iodoresiniferatoxin (I-RTX) was purchased from Tocris Cookson Inc. (Ellisville, MO). Ruthenium red was purchased from Sigma-Aldrech (St. Louis, MO). 2-Aminoethoxydiphenyl borate (2-APB) and 4-alpha-Phorbal 12,13-didecanoate (4-alpha PDD) were purchased from Enzo Life Sciences (Farmingdale, NY). Each of the following N-acyl amides were purchased from Cayman Chemical (Ann Arbor, MI): *N*-arachidonoyl ethanolamine (Anandamide; AEA), N-oleoyl ethanolamine (OEA), N-palmitoyl ethanolamine (PEA), *N*-linoleoyl ethanolamine (LEA), *N*-docosahexaenoyl ethanolamine (DEA), N-arachidonyl glycine (NAGly), and 2-AG All other reagent-grade *N*-acyl amides were synthesized in house as previously described (Tan et al., 2010). Unless otherwise indicated, all compounds were stored in 100% ethanol, however, assay solutions were made fresh in DMSO prior to every assay. Chemicals used for mass spectrometry were HPLC-grade and include methanol, water, ammonium acetate, and acetic acid. HPLC water and methanol were purchased from VWR International (Plainview, NY). Ammonium acetate and acetic acid were purchased from Sigma Aldrish (St. Louis, MO).

### Animals

All protocols were approved by the Indiana University Institutional Animal Care and Use Committee. Adult male Sprague–Dawley rats (Harlan, Indianapolis, IN USA) were used in all experiments. Rats were kept 4 to a cage in standard cages on a 12:12-h light–dark cycle. Food and water were available *ad libitum*. All subjects were adapted to the laboratory environment for a minimum of 2 weeks prior to use in our studies.

### Cell culture

Human embryonic kidney cells (HEK-293) stably expressing human TRPV1-4 and BV-2 cells were cultured as monolayers in T-75 flasks and expanded to passages 15–23. Cells were maintained in Dulbecco's minimum essential medium (DMEM) modified with non-essential amino acids, L-glutamine, and 2.2 g/L sodium bicarbonate and supplemented with 10% fetal bovine serum (FBS) and 1% penicillin-streptomycin (P/S). Cells were subcultured an average of three times a week with Trypsin-EDTA 1× and grown at 37°C in a humidified atmosphere of 5% CO2 in air; this was done 48 h prior to calcium assays.

### Calcium (Ca^2+^) imaging assay

The effect of the *N*-acyl amides on calcium mobilization was assessed using a high-throughput 96-wells plate assay adapted from our previous work (Rimmerman et al., 2008). TRPV1 (human), TRPV2 (mouse), TPRV3 (mouse), or TRPV4 (rat)-transfected HEK-293 (TRPV2 cells were a kind gift from Vincenzo DiMarco and the TRPV3 and 4 cells were a kind gift from Michael Caterina) cells were seeded on Cell BIND 96-well flat clear bottom black polystyrene microplates (Corning Life Sciences, Acton, MA) coated with 50 μg/mL poly-D-Lysine (PDL) under the cell culture hood at room temperature, allowing cells to attach to plates. Cells were grown to 75–85% confluence in a monolayer in the culture medium described above for 24–48 h. Prior to all experiments, cells were examined under the microscope to check for optimal confluency and normal morphology.

### Agonist activity calcium mobilization screens

Prior to loading the cells, the selective intracellular fluorescent probe for calcium, Fura-2-acetoxymethyl ester (Fura-2AM), was removed from –20°C and allowed to equilibrate to room temperature while protected from light. Cells were then loaded with 3 μM Fura-2AM at room temperature for 1 h, in HEPES-Tyrode buffer containing 0.05% w/v pluronic F-127 (Invitrogen). HEPES-Tyrode buffer was prepared with HEPES 0.025 mol, NaCl 0.14 mol, KCl 0.0027 mol, CaCl_2_ 0.0018 mol, MgCl_2_ 0.0005 mol, NaH_2_PO_4_ 0.0004 mol, and glucose 0.05 mol, and HEPES-Tyrode buffer used in calcium imaging assays was adjusted to pH 7.4 in order to avoid cellular ionic perturbation. After 1 h of Fura-2AM incubation, cells were washed twice and incubated in 175 μL of HEPES-Tyrode buffer for 20 min at room temperature. The plates were scanned for calcium activity using Flex Station II (Molecular Devices, Sunnyvale, CA) at 27°C. After 30 s of baseline recording, cells were challenged with 75 μL of buffer containing *N*-acyl amide solutions or positive control agonists capsaicin (TRPV1), 2-APB (TRPV2 and 3), 4α PDD (TRPV4), or PAF (BV2) which was first dissolved in DMSO or challenge compounds that were first dissolved in 100% DMSO at different concentrations and then added to buffer for a final DMSO concentration of 0.5% or vehicle (DMSO) alone using the automated fluidic module of Flex Station II.

### Antagonist activity screens

Similar to the agonist activity assay, cells were incubated at room temperature for 1 h with 3 μM Fura-2AM in HEPES-Tyrode buffer containing 0.05% w/v pluronic F-127. Cells were then washed twice and resuspended in 175 μL of HEPES-Tyrode buffer containing the 10 μM *N*-acyl amide mixture or individual compound (depending on the assay) which was first dissolved in 100% DMSO and added to the buffer to make a final concentration of DMSO of 0.5%. or vehicle (DMSO) alone and incubated for 20 min. These plates were then loaded into the FlexStationII and following 30 s of baseline recording, the pre-incubated cells were then challenged with 75 μL of HEPES-Tyrode buffer containing the corresponding positive control agonists capsaicin (TRPV1), 2-APB (TRPV2 and 3), or 4α PDD (TRPV4) which was first dissolved in DMSO. The plates were then scanned for calcium activity using Flex Station II (Molecular Devices, Sunnyvale, CA) at 27°C. The following positive control antagonists were used to compare the level of activity of N-acyl amide mixtures or individual lipids: Iodo-resiniferatoxin (I-RTX; TRPV1), Ruthenium Red (TRV2-4).

### *In vivo* induced paw inflammation

Male Sprague–Dawley rats remained in their home cage but were placed in the testing room for at least 1 h prior to injection to allow for acclimation to the novel environment. Rats were divided into two groups. Control groups received a 50 μL intraplantar injection of vehicle (saline) into the right hind paw and experimentally-treated animals received a 50 μl intraplantar injection of 3% carrageenan (CG) dissolved in saline into the right hind paw. All solutions of CG or saline were adjusted to pH 7.4.

### Paw inflammation assessment and tissue extraction

Paw inflammation was evaluated in all animals at 3 h following carrageenan injection by using calipers to measure dorsal-to-ventral paw thickness. Rats were euthanized by decapitation and brains removed, placed in Eppendorf tubes and flash-frozen in liquid nitrogen. After brains were frozen, they were quickly dissected and the striatum, thalamus, hippocampus, cerebellum, midbrain and brainstem were placed in individual ependorph tubes, and flash frozen in liquid nitrogen. Tissue samples were kept in a −80°C freezer until used for lipid extraction.

### Liquid chromatography/tandem mass spectrometry (LC/MS-MS)

Levels of each compound were analyzed by multiple reactions monitoring (MRM) mode using an applied Biosystems/MDS Sciex triple quadrupole mass spectrometer API 3000 (Foster City, CA) with electrospray ionization as previously described (Bradshaw et al., 2006; Rimmerman et al., 2008; Lee et al., 2010; Ho et al., 2012; Tortoriello et al., 2013). Samples were loaded using Shimadzu SCL10Avp auto-sampler and chromatographed on a 210 mm Zorbax Eclipse XDB-C18 reversed-phase HPLC column (3.5 μm internal diameter) maintained at 40°C. The flow rate was 200 μL/min achieved by a system comprised of a Shimadzu controller and two Shimadzu LC10ADvp pumps. The Shimadzu LC10ADvp HPLC pumps operated with a starting gradient of 0% mobile phase B which was increased to 100% before returning back to 0%. The mobile phase A consisted of 20/80 MeOH/water containing 1 mM ammonium acetate and mobile phase B consisted of 100% MeOH containing 1 mM ammonium acetate and 0.5% acetic acid.

### Data analysis

The analysis of calcium imaging experiments using 96-wells plate was as follows: after 30 s of baseline recording, cells were challenged with the drug, and calcium data per each well was collected every 5 s for a total scan time of 200 s and then calculated as the area under the curve (in relative florescence units) using Softmax Pro 5 integration functions (Molecular Devices, Union City, CA). Levels of calcium flux per each treatment group were collected from all repeats and data are presented as mean ± standard error of the mean (SEM) from at least three different experiments. Graph Pad Prism was used for further statistical analysis. Results were analyzed using One-Way ANOVA with Bonferroni's (calcium imaging data) or Fishers LSD (lipidomics) *post hoc* tests. EC_50_ are calculated by non-linear regression analysis using the equation for sigmoidal concentration-response curve in Graph Pad Prism 4.0. ^*^p-value of *p* < 0.05 or ^#^*p*-value of *p* < 0.01 are considered significant.

The 340/380 fluorescence intensity ratio of Fura2AM emission was collected every 5 s for a total scan time of 200 s. Calcium flux per each well was calculated as the area under the curve in relative fluorescence units using Softmax program integration functions. Graph Pad Prism was used for further statistical analysis. The number of repeats per each treatment group varies between 5 and 10 from at least three independent experiments. The majority of mixtures of *N*-acyl amides caused calcium mobilization that was significantly different from vehicle alone when analyzed with an alpha level of *p* < 0.05, however, the effect size was of a low magnitude. In an attempt to narrow down those groups that had the greatest magnitude effect, we set the alpha level to *P* < 0.01.

HPLC/MS-MS data was analyzed as previously described (Bradshaw et al., 2006; Rubio et al., 2007; Rimmerman et al., 2008; Lee et al., 2010; Tan et al., 2010). In brief, the detection of each analyte was based on fragmentation of the precursor ion to yield a daughter ion in the negative ion mode with MRM. The retention times of each of the analyzed compounds were compared to those obtained from their corresponding standards. The area under the curve for the appropriate compound was obtained. The amount of each compound was extrapolated from a calibration curve obtained from analyzing known concentration of synthetic standards and then corrected based up on the extraction efficiency. Final expression of total amounts are in mols/gram tissue (wet wt.). ANOVA analyses were made between 1 h post vehicle and carrageenan injection or 3 h post vehicle or carrageenan injection treatment groups. Individual ANOVAs were performed for each individual lipid family in that there was no a priori assumption that any one lipid specie would have an effect on another, nor would there be interactions between them.

## Results

### Calcium mobilization of N-Acyl amide families at TRPV1-4

The first set of experiments were aimed at screening mixtures of each of the *N*-acyl amide families from our unique library of compounds in each of the TRPV-HEK expression systems. Supplemental Table 1 identifies the complete list of compounds used in the screen and they are grouped as a structural “family” from the categorization of the amine as the grouping mechanism. As an example, all those *N*-acyl glycine molecules that are in the library are considered a unique “family” of compounds and a mixture of each at the same molarity was used as a single screening tool in each of the transfected cell lines. Each of the TRPV1-4 expression systems tested had at least one positive hit with an *N*-acyl amide mixture (**Table 2**). For agonist activity 4 different families drove calcium in TRPV1-HEK cells, TRPV2 had 2, TRPV3 had 1, and TRPV4 had 2. Whereas, none of the novel *N*-acyl amide mixtures induced calcium mobilization in non-transfected HEK-293 cells (Table 1).

**Table 1:** **Agonist activity of *N*-acyl amides at TRPV1-4**.

**Agonist activity**					
***N*-acyl amides (10 μM mixtures)**	**Non-transfected HEK-293**	**TRPV1**	**TRPV2**	**TRPV3**	**TRPV4**
*N*-acyl alanine	7.88 ± 3.50	16.71 ± 0.81	45.74 ± 5.58	14.89 ± 2.19	30.13 ± 3.94
*N*-acyl aspartic acid	11.97 ± 4.60	**95.13 ± 10.24**	6.41 ± 0.79	26.72 ± 4.11	25.58 ± 6.19
N-acyl beta-alanine	5.16 ± 3.76	22.78 ± 1.34	23.88 ± 5.21	21.18 ± 1.71	9.35 ± 3.29
N-acyl GABA	−8.15 ± 4.21	**153.08 ± 10.25**	23.58 ± 5.13	20.24 ± 2.76	16.00 ± 2.97
N-acyl glycine	−20.47 ± 8.29	**85.61 ± 16.80**	22.10 ± 5.43	12.14 ± 2.98	32.03 ± 4.23
N-acyl isoleucine	−9.52 ± 3.25	20.20 ± 2.71	31.16 ± 7.62	9.83 ± 2.23	17.37 ± 4.39
N-acyl leucine	2.78 ± 1.79	10.00 ± 1.92	16.99 ± 2.19	−2.00 ± 0.32	4.20 ± 1.83
N-acyl methionine	−10.34 ± 4.65	17.02 ± 2.04	30.67 ± 5.03	28.65 ± 4.82	30.46 ± 4.97
N-acyl phenylalanine	−15.04 ± 3.00	0.68 ± 1.75	−5.90 ± 1.45	−7.55 ± 1.22	24.13 ± 3.54
N-acyl proline	−8.21 ± 2.41	28.34 ± 5.73	**73.35 ± 2.20**	23.92 ± 3.72	23.84 ± 4.95
N-acyl serine	−9.98 ± 6.27	**128.76 ± 29.90**	2.87 ± 2.46	20.56 ± 1.96	6.28 ± 1.76
N-acyl threonine	−0.47 ± 2.52	19.40 ± 1.01	1.57 ± 1.37	12.66 ± 1.05	20.40 ± 2.27
N-acyl tryptophan	11.39 ± 2.55	19.33 ± 2.81	3.94 ± 1.18	13.46 ± 1.49	**75.59 ± 7.79**
N-acyl tyrosine	−13.72 ± 5.83	39.05 ± 2.61	**74.78 ± 15.21**	**43.61 ± 2.42**	**55.59 ± 7.79**
N-acyl valine	−5.14 ± 4.69	26.63 ± 3.63	9.01 ± 1.70	13.42 ± 3.79	23.86 ± 4.27
DMSO	−5.46 ± 2.93	8.10 ± 0.75	−1.59 ± 1.68	−0.78 ± 1.36	2.14 ± 0.98
Capsaicin (10 μM)		**294.67 ± 31.31**			
NADA (10 μM)	−11.92 ± 2.74	**220.14 ± 25.25**			
2-APB			**238.24 ± 13.38**	**121.6 ± 3.48**	
4α-PDD					**76.92 ± 10.99**

**Figure 2:**
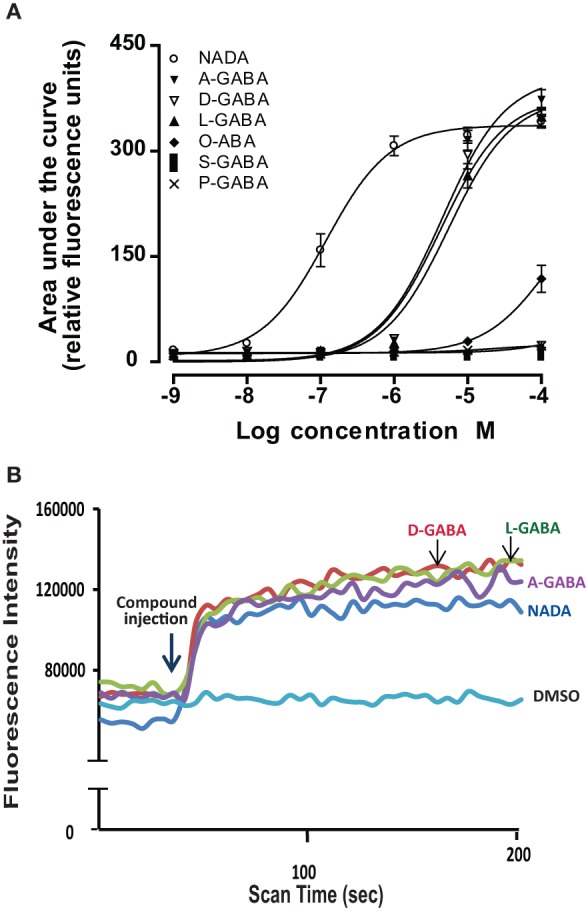
**Individual *N*-acyl GABA species drive calcium in TRPV1-transfected HEK cells**. **(A)** Concentration curves of individual *N*-acyl GABA: *N*-docosahexaenoyl GABA (D-GABA), *N*-arachidonoyl GABA (A GABA), *N*-linoleoyl GABA (L GABA), *N*-oleoyl GABA (O GABA), *N*-palmitoyl GABA (P GABA) and *N*-stearoyl GABA (S GABA) compared to the potent TRPV1 agonist, N-arachidonoyl dopamine (NADA). **(B)** Flourescence intensity that is an indication of calcium mobilization as measured by the ratiometric calcium-sensitive dye FURA2am in response to D-GABA, A GABA, L GABA, NADA, and the vehicle, DMSO. Data were generated in a Flexstation II (Molecular Devices).

Far left column lists the N-acyl amide family or control compound that was used as the challenge compounds for each screen. Subsequent columns contain the data from each individual TRPV-HEK expression system as well as the untransfected HEK cells. Values shown are the change in relative fluorescence units of Fura2AM from the 30 s baseline and represent the area of the curve of analysis of 190 s post injection (see example on Figure 2). The overwhelming majority of N-acyl amide mixtures caused significant increases in baseline calcium at p = 0.05 levels over that of the DMSO controls, which were the equivalent to zero. In order to streamline the screening process, we chose to focus on those differences that were only p ≤ 0.01. Those values in bold black are significantly different from those of the vehicle injection at p ≤ 0.01.

### Calcium mobilization of individual N-Acyl amides in TRPV1-4 HEK expression systems

**Figure 3:**
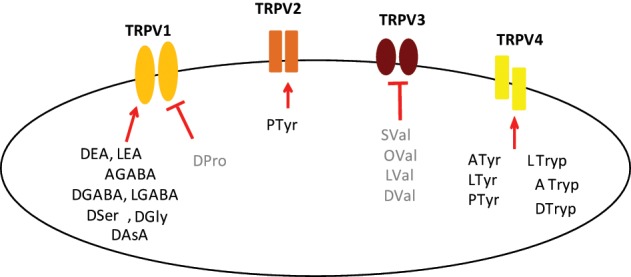
**Composite for all individual *N*-acyl amides tested here with activity at TRPV1-4**. This cartoon illustrates N-acyl amides that were shown to be active as agonists (Bold Black; arrow) and antagonists (Bold Gray; bar) in each of the TRPV1-4 expression systems, which are represented simply as the individual receptor. Agonists at TRPV1: *N*-docosahexaenoyl GABA (DGABA), *N*-arachidonoyl GABA (AGABA), *N*-linoleoyl GABA (LGABA); *N*-docosahexaenoyl serine (DSer), *N*-docosahexaenoyl glycine (DGly), *N*-docosahexaenoyl aspartic acid (DAsA), *N*-docosahexaenoyl ethanolamide (DEA), *N*-linoleoyl ethanolamide (LEA); Antagonists at TRPV1: *N*-docosahexaenoyl proline (DPro); Agonists at TRPV2: N-palmitoyl tyrosine (PTyr); Antagonists at TRPV3: *N*-docosahexaenoyl valine (DVal), *N*-linoleoyl valine (LVal), *N*-oleoyl valine (Oval), *N*-stearoyl valine (SVal); Agonists at TRPV4: *N*-arachidonoyl tyrosine (ATyr), *N*-linoleoyl tyrosine (LTyr); PTyr; *N*-docosahexaenoyl tryptophan (DTryp), *N*-arachidonoyl tryptophan (ATryp), *N*-linoleoyl tryptophan (LTryp).

In a second level of analysis, each individual member of the *N*-acyl amide mixtures that stimulated calcium mobilization in the first screen was tested separately in the same TRPV1-4 HEK/Fura-2AM system. Figure 2 shows an example case of individual *N*-acyl GABA compounds being screened in the TRPV1-HEK system. These experiments with the *N*-acyl GABAs showed a structure-activity relationship for *N*-docosahexaenoyl GABA (D-GABA), *N*-linoleoyl GABA (L-GABA), and *N*-arachidonoyl GABA (A-GABA) all causing significant calcium mobilization; therefore we extended the analysis to include full concentration curves for all *N*-acyl-GABAs. D-GABA, L-GABA, and A-GABA significantly induced concentration-dependent activation of TRPV1 and that each reached an asymptote equivalent to NADA (Figure 2A). Similar to NADA, D-GABA, L-GABA, and A-GABA induced an immediate, sustained increase of calcium (Figure 2B). Supplementary Figures 1–6 show additional data on individual *N*-acyl amide activity in TRPV1-4 HEK systems. These data revealed an additional 5 *N*-acyl amides at TRPV1 (*N*-docosahexaenoyl serine, *N*-docosahexaenoyl glycine, *N*-docosahexaenoyl aspartic acid, *N*-docosahexaenoyl ethanolamide, *N*-linoleoyl ethanolamide), 1 at TRPV2 (*N*-palmitoyl tyrosine), none above the statistical threshold at TRPV3 and 6 at TRPV4 (*N*-arachidonoyl tyrosine, *N*-linoleoyl tyrosine, *N*-palmitoyl tyrosine, *N*-docosahexaenoyl tryptophan, *N*-arachidonoyl tryptophan, *N*-linoleoyl tryptophan). These findings are summarized as a cartoon in Figure 3.

### Antagonist activity at TRPV1-4 by N-Acyl amides

*N*-acyl amide mixtures listed in Supplemental Table 1 were screened as antagonists at TRPV1-4. As with the agonist screens, we used a known antagonist for each of the TRPV expression systems as our antagonist positive control and challenged the TRPV-HEK expression systems with the same known agonist as in the agonist screens (see Materials and Methods for list of compounds used). Table 2 shows the positive hits for each TRPV expression system. The majority are those that also showed agonist activity also showed antagonist activity (e.g., *N*-acyl GABA mixture is a strong agonist/antagonist at TRPV1), which suggests a likely competitive inhibition. Therefore, we focused our efforts for identifying antagonists to those hits that did not have any agonist activity, which singled out the *N*-acyl proline mixture at TRPV1 and the *N*-acyl leucine and *N*-acyl valine mixtures at TRPV3.

**Table 2:** **Antagonist activity of *N*-acyl amides at TRPV1-4**.

**Antagonist activity**				
**N-acyl amides (10 μ M mixtures)**	**TRPV1 (+ CAP)**	**TRPV2 (+ 2-APB)**	**TRPV3 (+ 2-APB)**	**TRPV4 (+ 4α-PDD)**
N-acyl alanine	155.12 ± 10.33	267.43 ± 14.53	74.55 ± 4.34	87.46 ± 8.03
N-acyl aspartic acid	187.20 ± 11.78	251.09 ± 12.01	65.74 ± 13.38	83.25 ± 8.34
N-acyl beta-alanine	166.84 ± 13.52	239.05 ± 25.01	92.69 ± 9.95	91.59 ± 5.26
N-acyl GABA	**87.22 ± 11.12**	277.24 ± 22.89	163.03 ± 15.23	94.24 ± 5.71
N-acyl glycine	151.43 ± 10.3	255.01 ± 16.16	123.01 ± 10.80	85.11 ± 7.15
N-acyl isoleucine	185.08 ± 5.76	285.96 ± 30.44	62.73 ± 3.80	97.22 ± 5.46
N-acyl leucine	179.18 ± 13.56	235.66 ± 22.26	**51.37 ± 3.10**	80.25 ± 6.18
N-acyl methionine	158.93 ± 14.24	237.28 ± 18.32	65.15 ± 5.74	91.50 ± 9.87
N-acyl phenylalanine	180.70 ± 10.01	260.15 ± 28.61	75.64 ± 22.64	81.56 ± 4.54
N-acyl proline	**95.98 ± 21.26**	**−34.61 ± 26.70**	142.49 ± 6.48	94.16 ± 11.34
N-acyl serine	196.28 ± 6.53	234.06 ± 22.36	69.65 ± 5.10	94.83 ± 5.23
N-acyl threonine	197.53 ± 7.51	210.15 ± 17.10	81.18 ± 5.96	83.89 ± 7.96
N-acyl tryptophan	201.35 ± 3.26	263.71 ± 24.69	61.91 ± 6.13	**−99.49 ± 38.83**
N-acyl tyrosine	156.56 ± 15.15	**−116.42 ± 13.94**	58.78 ± 5.40	**−124.36 ±, 22.53**
N-acyl valine	174.62 ± 11.94	240.61 ± 26.79	**39.73 ± 4.16**	37.05 ± 7.37
DMSO	195.69 ± 16.42	253.67 ± 39.42	95.73 ± 7.01	93.92 ± 11.55
I-RTX (20 nM)	**−22.15 ± 5.80**			
Rethenium red (30 μM)		**64.83 ± 4.51**	**41.33 ± 3.75**	**−85.52 ±, 34.61**

*Far left column lists the N-acyl amide family or control compound that was used as the pre-incubated compounds for each screen. Challenge compounds are listed under the title of the TRPV-HEK expression system. Values shown are the data from the change in relative fluorescence units of Fura2AM from the 30 s baseline and represent the area of the curve of analysis of 190 s post injection of the positive control challenge compound. Those values in bold black are significantly different from those of the vehicle injection p ≤ 0.01*.

### Antagonist activity on calcium mobilization of individual N-Acyl amides in TRPV1 and TRPV3 HEK expression systems

Of particular interest in the category of the TRPV1 screen was the *N*-acyl proline mixture. This mixture had no agonist activity and a screen of the individual members revealed that *N*-docosahexaenoyl proline is a potent TRPV1 inhibitor (Supplemental Figure 7). Likewise, the *N*-acyl valine mixture had no agonist activity at TRPV3, however, there were 4 individual *N*-acyl valine compounds that act as antagonists (*N*-docosahexaenoyl valine, *N*-linoleoyl valine, *N*-oleoyl valine, *N*-stearoyl valine; Supplemental Figure 8), whereas, the activity of individual *N*-acyl leucine species were not significant. All of the individual *N*-acyl amides that activate or inhibit the TRPV1-4 receptors are summarized in Figure 3.

### Lipidomics of 6 distinct brain regions after peripheral inflammation reveal novel endogenous N-Acyl amide regulation

To address one potential aspect of functional relevance of *N*-acyl amide signaling, we performed a series of targeted lipidomics screens of 75 *N*-acyl amides via HPLC-MS/MS on 6 distinct areas of the brain (striatum, hippocampus, cerebellum, thalamus, midbrain, and brainstem) in order to survey *N*-acyl amide regulation during an acute inflammatory stimulus. Using the intraplantar carrageenan peripheral inflammation model, we induced an acute inflammatory response and measured changes in brain levels of *N*-acyl amides. Supplemental Figure 9 shows that the levels of edema in the injected paw were significantly greater than vehicle injected at both 1 and 3 h post injection. A majority of all the lipids measured were detected in each of brain areas surveyed and each lipid was found in pmol/gram levels (Supplemental Figure 9 shows matching chromatograms from standards and extracts for 3 *N*-acyl GABA species; Supplemental Table 2 shows all the averaged values for each of the lipids detected in each brain area). Analysis of all lipids in each brain area and comparisons among the four treatment groups was multi-variant. Given that here were four treatment groups and an n of 8 per treatment group, mass spectrometric analysis yielded an enormous quantity of data totaling 14,400 data points for these six brain areas tested (Data shown in Supplemental Table 2). Here, we provide an example of the analysis of those measurements in the hippocampus and striatum.

Out of the 75 spectra analyzed, 48 analytes were detected in hippocampal samples, whereas 57 were detected in striatal samples. Two different levels of analyses were performed. The first level was to compare Veh-1H to CG 1H and then the second level of analysis was to compare Veh-3H and CG-3H. To simplify the analysis into more manageable units, we compared only those lipids within an *N*-acyl amide family in a single ANOVA. For example, only *N*-acyl ethanolamides were compared to the same *N*-acyl ethanolamides. Future studies are aimed at using these data sets in a modeling system to determine if there are predictive directions of types of *N*-acyl amides or their fatty acid derivatives in a particular direction, but that is beyond the scope of the analysis here.

Table 3 summarizes the results of the 34 *N*-acyl amides that were shown to be modified at either 1 or 3 h post injection as a function of each individual brain area. Of the 12 lipids that were modified (either increasing or decreasing) at the 1 h time point, 6 were in found in the hippocampus and 9 of the 12 significantly decreased. This is in stark contrast to the changes in the levels of *N*-acyl amides in the brain 3 h after carrageenan injections wherein all the *N*-acyl amides that were modified by the treatment were significantly higher than those with vehicle injection. Whereas the most differences measured at 1 h post carrageenan were in the hippocampus, the area of the brain with the most dramatic changes in *N*-acyl amides at 3 h post carrageenan were in the striatum and cerebellum. Notably, all brain areas tested had significant increases in *N*-acyl ethanolamides and three of the six (striatum, hippocampus, and cerebellum) all had significant increases in *N*-acyl glycines. Of the 34 *N*-acyl amides that were up or down regulated with peripheral inflammation, 10 showed activity at TRPVs in the assays discussed above and are denoted in gray in Table 3.

**Table 3:**
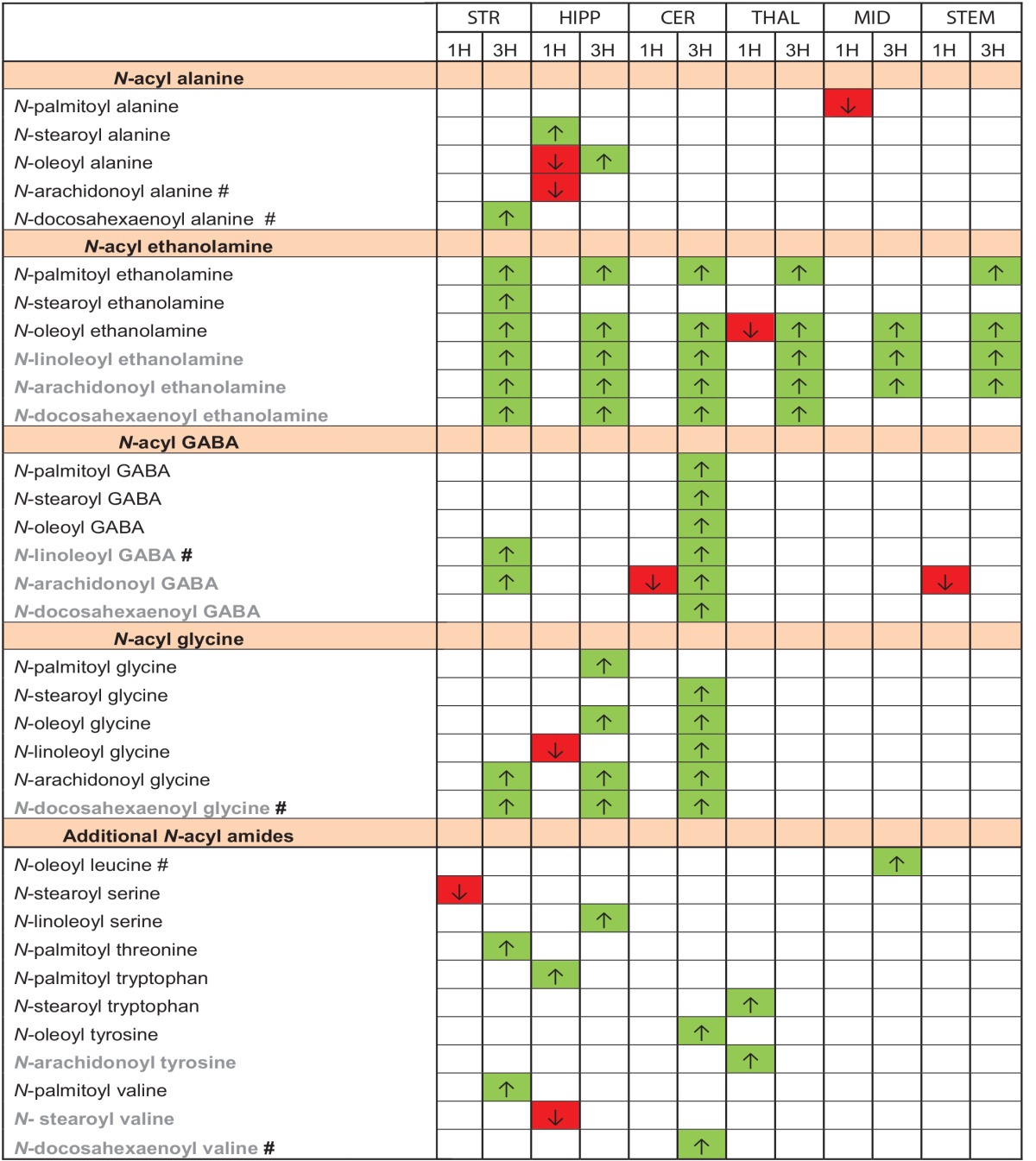
**Effects on *N*-acyl amide lipidomics profile in 6 brain areas 1 and 3 h post carrageenan**.

*These are the summary data of those N-acyl amides that were significantly modified (increased or decreased) from the levels measured in the vehicle control and do not represent all lipids tested. All averaged values for all lipids tested are shown in Supplemental Table 2. Six brain areas (STR, striatum; HIPP, hippocampus; CER, cerebellum; THAL, thalamus; MID, midbrain; STEM, brainstem) are depicted in individual columns further separated by the treatment groups 1 hour (1H) and 3 hour (3H) post carrageenan. Those in green with an up arrow showed significant increases from control injections, whereas, those in red with down arrows showed significant decreases from control injections p = 0.05. Those in gray are N-acyl amides that activated on of the TRPV systems tested here. ^#^Denotes that these compounds had not been previously identified in mammalian brain*.

### Calcium mobilization by N-Acyl amides in BV-2 microglial cells

Activation and mobilization by microglia occurs not only during times of mechanical and pathogen-induced tissue damage, but also in times of acute pain (Ji et al., 2013). To test the hypothesis that *N*-acyl amides would drive calcium mobilization in microglia, we screened *N*-acyl amide mixtures in the microglial cell line BV2. Table 5 shows that 5 different *N*-acyl amide family mixtures drive calcium mobilization in BV2 cells, whereas 1 mixture (*N*-acyl phenylalanines) caused a significant depression of the baseline calcium signaling. We chose one mixture, the *N*-acyl GABA lipids to assess activity by individual lipid species. Supplemental Figure 10 shows that there is a structure activity relationship in the *N*-acyl GABA family. Importantly, however, there was no calcium mobilization by capsaicin (Table 4) suggesting that *N*-acyl GABA lipids are likely acting through an alternative receptor.

**Table 4:** **Agonist effects of *N*-acyl amide mixtures on calcium mobilization in BV-2 microglia**.

**N-acly amide mixtures 10 μ M**	**BV-2 Microglia**
N-acyl alanine	**69.48 ± 3.18**
N-acyl aspartic acid	12.73 ± 0.82
N- acyl beta-alanine	15.33 ± 1.16
N- acyl GABA	**119.56 ± 2.72**
N- acyl glycine	22.91 ± 1.32
N-acyl isoleucine	8.31 ± 1.17
N-acyl leucine	56.47 ± 1.62
N-acyl methionine	33.00 ± 1.84
N-acyl phenylalanine	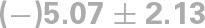
N-acyl proline	**102.66 ± 2.04**
N-acyl serine	12.41 ± 1.35
N-acyl threonine	12.27 ± 1.00
N-acyl tryptophan	4.83 ± 1.04
N-acyl tyrosine	19.74 ± 1.24
N-acyl valine	**61.33 ± 2.17**
DMSO	9.09 ± 0.84
Capsaicin (500 nM)	9.60 ± 2.54
PAF	**49.60 ± 2.71**
Inonomycin	**223.92 ± 5.81**

Far left column lists the N-acyl amide family or control compound that was used as the challenge compounds for each screen. Values shown are the change in relative fluorescence units of Fura2AM from the 30 s baseline and represent the area of the curve of analysis of 190 s post injection (see example on Figure 2. Those values in bold black are significantly higher from those of the vehicle injection, whereas those in bold gray are significantly lower p ≤ 0.01.

## Discussion

Lipidomics as a field aims to both identify the full complement of biologically active lipids as well as their functional relevance. Here, we have combined a bottom–up approach targeting activity of novel *N*-acyl amide lipids at specific TRPV1-4 receptors and BV2 microglia with a top–down approach of screening the production or metabolism of these lipids in the brain in a model of peripheral inflammation and pain. Together these approaches provide a powerful set of tools that enabled us to discover additional endogenous ligands for TRPV1-4 and microglia as well as determine how these ligands are regulated in the CNS with inflammation, thereby generating a novel hypothesis for the activation and regulation of lipid signaling molecules and their receptors.

### Bottom-up lipidomics of N-Acyl amides at TRPV1-4 and BV-2 microglia

TRPV receptor activation and regulation as part of the processing of nociceptive information in the periphery and the CNS is not fully understood. Here, we identified 20 novel *N*-acyl amides as putative ligands for TRPV1-4 channels. These data add to the growing literature that activation of TRP channels follows an opportunistic strategy in which, a wide range of structurally similar endogenous lipids are capable of driving calcium mobilization through these channels (Movahed et al., 2005; Bradshaw et al., 2013). An interesting example of this opportunistic strategy is the evidence here demonstrating that TRPV1 is activated by eight additional novel *N*-acyl amides, including *N*-docosahexaenoyl ethanolamine, which including the previous work by Movahed et al. (2005) brings the number of lipids in the N-acyl ethanolamine family alone up to at least 5. One difference between the two studies was the potency of OEA at TRPV1, which was higher than 10 μM in our hands, therefore, not further investigated here. However, this discrepancy may be due to the very different assay conditions between the two studies. We were able to detect all of these novel TRPV1 agonists except *N*-docosahexaeonyl aspartic acid in the brain. These data support the existing hypothesis that the primary endogenous TRPV1 ligands are long-chain, unsaturated acyl-amides (e.g., AEA; NADA; OLDA) and extends the number of endogenous *N*-acyl amide TRPV1 ligands to at minimum 13. Even though we were unable to test all the individual lipids in our screen at each of the TRPVs tested here, of those that were individually screened the vast majority did not activate TRPV1, suggesting that it is not simply a response to long-chain *N*-acyl amides of any structure. This finding replicates an earlier finding that NAGly and the oxidized AGABA (AGABA-OH) had no activity at TRPV1 (Barbara et al., 2009). Therefore, the SAR of activity is specific to the oxidation level of the same molecule. In addition, the one antagonist that was identified here, *N*-docosahexaenoyl proline, is also a long-chain fatty acid amide, providing further evidence of the structural properties of those molecules that successfully interact with the TRPV1 receptor. Therefore, while our and previous data support the hypothesis that TRPV1 is an opportunistic receptor that preferentially is activated by long-chain, unsaturated acyl-amides, it is not activated by all of such molecules in this category.

Similar results were seen with the identification of TRPV4 activators in that of the two *N*-acyl amide mixtures that were hits for agonist activity, three individual members of each were shown to activate the receptor. This was not the case, however, for activity at TRPV2 and TRPV3. Wherein the *N*-acyl proline mixture had the most activity at TRPV2, none of the individual members had a significant effect. While *N*-palmitoyl tyrosine did have a significant effect on calcium mobilization, it was relatively weak compared to the *N*-acyl tyrosine mixture at the same molarity. Likewise, the *N*-acyl tyrosine mixture had the strongest agonist effect at TRPV3, yet none of the individual members showed significant activity. Intriguingly, each of these mixtures also caused antagonist activity in the same expression systems. We hypothesize that there are allosteric effects of individual lipids on members of the same *N*-acyl amide family that would drive one member to have a higher potency than another. This hypothesis will be tested in future studies.

*N*-acyl amide activity in BV2 microglia also showed similar patterns to those of the TRPVs tested here. 4 of the 5 *N*-acyl amide mixtures that caused calcium mobilization in these BV2 microglia also activated different TRPVs here, with the only exception being *N*-acyl alanine, which did have some activity at TRPV2, though not at the 0.01 *p*-value; therefore, this was not pursued in these studies. Further studies are aimed at determining if the N-acyl alanines are active at additional TRPs. As mentioned previously, however, there was not calcium mobilization with capsaicin in these BV2 cells, suggesting that none of these responses would be through TRPV1. Study of TRP receptor function in microglia is a small but growing field. Although the presence of TRPV1-4 on BV-2 microglial cells has not been fully confirmed, there exists plentiful evidence to suggest a role for TRPVs in microglial signaling. Hassan and colleagues measured the rate of phagocytosis in cultured microglial cells by quantifying the ingestion of fluorescently labeled latex beads. The phytocannabinoid cannabidiol (CBD) boosted the rate of phagocytosis, specifically, 10 μM CBD increased phagocytosis by 175% over vehicle control. BV-2 microglia do not express the cannabinoid receptors CB_1_ or CB_2_, thus the effect was not mediated by CB receptor signaling. Pretreating with pertussis toxin, which blocks GPCRs, had no effect on CBD's actions, ruling out GPCR signaling pathways as mediators of this effect. In addition to increasing phagocytosis, CBD caused calcium influx into the microglial cells. The calcium influx was abolished by ruthenium red, a non-selective TRP channel antagonist. Additionally, ruthenium red prevented CBD from increasing phagocytosis. The effects on individual TRPV channels were then investigated: TRPV1 was implicated, as treatment with capsazepine blocked the increase in phagocytosis. Without CBD treatment, the TRPV2 agonist probenecid dose-dependently enhanced phagocytosis *in-vitro*. RT-PCR revealed that TRPV2 mRNA is detectable in untreated BV-2 cells (Hassan et al., 2014).

Immunocytochemistry has shown TRPV1 to be present in rat primary microglial cell lines. However, care must be taken in interpreting these results as knock-out controls are not shown and TRPV1 antibodies can produce artifactual staining. RT-PCR showed that TRPV1 mRNA was present on rat microglia, but not on astrocytes. Interestingly, exposure to TRPV1 agonists like capsaicin-induced apoptosis in these cultured microglial cells. These effects were inhibited by capsazepine, indicating a role for TRPV1 in regulating apoptosis in microglia (Kim et al., 2006). RT-PCR also showed TRPV1 mRNA expression in rat retinal microglial cultures, where it may possibly have a role in regulating intra-ocular pressure (Sappington and Calkins, 2008).

Eight out of 20 of the novel *N*-acyl amides identified here as TRPV1-4 activators are conjugates of docosahexaenoic acid, which is an omega-3 fatty acid. This is particularly interesting in the context of the recent focus in human nutrition on this class of lipids. Links between omega-3 fatty acids and pain associated with inflammation have been shown and studied for decades, however, the exact molecular mechanism remains elusive (for rev see Tokuyama and Nakamoto, 2011). Resolvin molecules are clearly part of the answer (Ji et al., 2011); however, their rapid degradation that perhaps leads to a lack of data on their endogenous production provides a roadblock in our understanding of how these molecules work *in vivo*. In our own hands, we have observed that *N*-docosahexaenoyl amides are significantly more stable and can be readily measured in tissue. We propose that these *N*-docosahexaenoyl amides may even act as more stable precursor molecules for the more reactive and fast-lived resolvins.

Unlike the resolvin species of lipids, *N*-acyl amides in brain are relatively straightforward to measure using methanolic extractions, partial purification on C18 solid phase extraction columns and HPLC-MS/MS analysis (Bradshaw et al., 2006; Rimmerman et al., 2008; Bradshaw et al., 2009; Smoum et al., 2010). We recently identified 47 of those measured here in drosophila, demonstrating that these simple bioactive lipids are not unique to mammals or the brain (Tortoriello et al., 2013). That nearly a third of those measured here were modified by peripheral inflammation suggests that they are of the type of lipids that are rapidly made on demand and are likely part of the myriad of signaling systems that are engaged during both a stress-inducing and painful event.

### Top–down lipidomics of N-Acyl amides in the CNS

Carrageenan injections into the paw are a reliable model of acute inflammation that is associated with thermal and mechanical allodynia and hyperalgesia as well as the suite of learning a motivated behaviors associated with the response to noxious stimuli (Fecho et al., 2005). Here, we demonstrate a wide-range of regulation of *N*-acyl amides in six different regions of the brain; however, we will focus our discussion on the hippocampus and the striatum in that they showed the most dynamic changes in *N*-acyl amides with peripheral inflammation. It is important to note that they both play important and evolving roles in pain and inflammation.

Both the striatum and hippocampus have been heavily implicated as CNS regions involved in pain processing and perception (Malow and Olson, 1981; Mcewen, 2001; Ploghaus et al., 2001; Ferre et al., 2009; Barcelo et al., 2012), and our study confirms that there are changes in the production of these putative *N*-acyl amide signaling molecules in the striatum and hippocampus due to inflammatory pain. However, one of the major limitations of this study is the heterogeneity of these brain areas. While both regions are discrete and easy to dissect from the brain, both the striatum and hippocampus are also responsible for a host of functions. The hippocampus is particularly noted for its role in consolidation of long-term memories and spatial memory in rodents. Constant dendritic remodeling and neurogenesis make the hippocampus capable of learning new associations such as avoidance behaviors to painful stimuli (Ploghaus et al., 2001). The hippocampus is also very susceptible to glucocorticoids for instance those released in response to a painful, stressful situation. McEwen hypothesizes that the neurochemical and morphological changes in the hippocampus may cause changes in chronic pain perception (Mcewen, 2001). Indeed, patients with effectively managed chronic pain versus those whose pain persists despite treatment have an increased pain threshold and are able to more sensitively discriminate between different levels of pain (Malow and Olson, 1981).

Similarly, the striatum is involved in regulating a variety of processes. The striatum is best known for its role in planning and modulating motor movement by integrating sensory and motor information. However, parts of the striatum respond exclusively to noxious stimuli (Barcelo et al., 2012). While dopaminergic neurons are densely expressed in the striatum and implicated in pain processing, CB_1_ receptors form heteromers with D_2_ A_2A_, and μ opioid receptors indicating potential novel mechanisms of endocannabinoid function in the striatum beyond retrograde signaling and inhibition of neurotransmitter release (Ferre et al., 2009). Therefore, increases in AEA shown here may play a role in the CB_1_ response as well as in the TRPV1 response.

## Conclusions

These studies represent a novel direction in ion channel research in the CNS. Blending our knowledge of lipidomics of *N*-acyl amides and TRPV channel activity has allowed us to discover a connection between the two of which the depth of has yet to be tested. We propose that this is merely the beginning of our understanding of how these systems work in concert and hypothesize that the co-evolution of *N*-acyl amides and ion channels plays a major role in a wide range of cellular signaling beyond the mammalian brain.

### Conflict of interest statement

The authors declare that the research was conducted in the absence of any commercial or financial relationships that could be construed as a potential conflict of interest.
