# Supplementary material for: Novel endogenous N-acyl amides activate TRPV1-4 receptors, BV-2 microglia, and are regulated in brain in an acute model of inflammation
Source: Front Cell Neurosci. 2014 Aug 1;8:195. doi: 10.3389/fncel.2014.00195 (PMC4118021; doi:10.3389/fncel.2014.00195)
Supplement: Supplementary file 1 [file DataSheet1.PDF]

***N*-Acyl ethanolamine**	***N*-Acyl GABA**	***N*-Acyl phenylalanine**	***N*-Acyl tyrosine**
N-Palmitoyl ethanolamine	*N*-Palmitoyl GABA	*N*-Palmitoyl phenylalanine	*N*-Palmitoyl tyrosine
N-Stearoyl ethanolamine	*N*-Stearoyl GABA	*N*-Stearoyl phenylalanine	*N*-Stearoyl tyrosine
N-Oleoyl ethanolamine	*N*-Oleoyl GABA	*N*-Oleoyl phenylalanine	*N*-Oleoyl tyrosine
N-Linoleoyl ethanolamine	*N*-Linoleoyl GABA	*N*-Linoleoyl phenyalanine	*N*-Linoleoyl tyrosine
N-Arachdonoyll ethanolamine	*N*-Arachidonoyl GABA	*N*-Arachidonoyl phenlalanine	*N*-Arachidonoyl tyrosine
N-Docosahexaenoyl ethanolamine	*N*-Docosahexaenoyl GABA	*N*-Docosahexaenoyl phenylalanine	*N*-Docosahexaenoyl tyrosine
***N*-Acyl leucine**	***N*-Acyl isoleucine**	***N*-Acyl serine**	***N*-Acyl alanine**
N-Palmitoyl leucine	*N*-Palmitoyl Isoleucine	*N*-Palmitoyl serine	*N*-Palmitoyl alanine
N-Stearoyl leucine	*N*-Stearoyl isoleucine	*N*-Stearoyl serine	*N*-Stearoyl alanine
N-Oleoyl leucine	*N*-Oleoyl isoleucine	*N*-Oleoyl serine	*N*-Oleoyl alanine
N-Linoleoyl leucine	*N*-Linoleoyl isoleucine	*N*-Linoleoyl serine	*N*-Linoleoyl alanine
N-Docosahexaenoyl leucine	*N*-Docosahexaenoyl isoleucine *	*N*-Arachidonoyl serine	*N*-Docosahexaenoyl alanine
***N*-Acyl proline**	***N*-Acyl methionine**	*N*-Docosahexaenoyl serine	***N*-Acyl beta-alanine**
N-Palmitoyl proline	*N*-Palmitoyl methionine	***N*-Acyl aspartic acid**	*N*-Linoleoy beta-alanine
N-Stearoyl proline	*N*-Stearoyl methonine	*N*-Palmitoyl aspartic acid	*N*-Palmitoyl beta-alanine
N-Oleoyl proline	*N*-Oleoyl methionine	*N*-Stearoyl aspartic acid	*N*-Stearoyl beta-alanine
N-Linoleoyl proline	*N*-Linoleoyl methionine	*N*-Oleoyl aspartic acid	*N*-Oleoyl beta-alanine
N-Arachidonoyl proline	*N*-Arachdonoyl methionine	*N*-Linoleoyl aspartic acid	***N*-Acyl threonine**
N-Docosahexaenoyl proline	*N*-Docosahexaenoyl methionine	***N*-Acyl valine**	*N*-Palmitoyl threonine
***N*-Acyl tryptophan**	***N*-Acyl glycine**	*N*-Palmitoyl valine	*N*-Stearoyl threonine
N-Palmitoyl tryptophan	*N*-Palmitoyl glycine	*N*- Stearoyl valine	*N*-Oleoyl threonine
N-Stearoyl tryptophan	*N*-Stearoyl glycine	*N*-Oleoyl valine	
N-Oleoyl tryptophan	*N*-Oleoyl glycine	*N*-Nervonoyl valine	
N-Linoleoyl tryptophan	*N*-Nervonoyl glycine	*N*-Linoleoyl valine	
N-Arachidonoyl tryptophan	*N*-Arachidonoyl glycine	*N*-Docosahexaenoyl valine	
N-Docosahexaenoyl tryptophan	*N*-Docosahexaenoyl glycine		

Supplemental Table 2

			STR			HIPP			CER			THAL			MID			STEM	
	Group		Mean	SE		Mean	SE		Mean	SE		Mean	SE		Mean	SE		Mean	SE
***N*-acyl alanine**		N			N			N			N			N			N		
N-palmitoyl alanine	Veh 1 hr	8	3.64E-11	2.45E-12	8	2.65E-11	1.91E-12	8	1.65E-11	4.47E-12	8	6.44E-12	1.06E-12	8	3.37E-11	1.90E-12	8	1.06E-11	1.85E-12
	Veh 3 hr	8	3.48E-11	1.22E-12	8	2.46E-11	2.95E-12	8	1.27E-11	1.78E-12	8	6.39E-12	9.36E-13	8	2.44E-11	1.57E-12	8	1.26E-11	1.41E-12
	CG 1 hr	5	3.31E-11	2.02E-12	8	2.19E-11	1.68E-12	8	1.16E-11	7.13E-13	8	7.13E-12	8.64E-13	8	2.61E-11	2.57E-12	8	1.49E-11	2.79E-12
	CG 3 hr	8	3.62E-11	2.04E-12	7	2.79E-11	3.43E-12	8	1.28E-11	8.84E-13	8	5.64E-12	7.03E-13	8	2.75E-11	2.04E-12	7	1.66E-11	4.87E-12
N-stearoyl alanine	Veh 1 hr	7	3.69E-11	3.23E-12	7	1.83E-11	1.21E-12	8	3.74E-11	1.76E-12	8	2.24E-11	3.29E-12	8	4.62E-11	8.01E-12	8	1.54E-11	2.09E-12
	Veh 3 hr	7	3.33E-11	1.33E-12	8	2.26E-11	3.21E-12	8	3.52E-11	2.51E-12	8	2.52E-11	3.79E-12	8	3.31E-11	1.11E-11	8	1.94E-11	2.94E-12
	CG 1 hr	7	3.12E-11	4.09E-12	8	2.85E-11	5.80E-12	8	3.25E-11	1.98E-12	8	2.01E-11	2.29E-12	8	3.20E-11	4.39E-12	8	1.63E-11	3.75E-12
	CG 3 hr	8	3.14E-11	4.92E-12	6	2.21E-11	3.50E-12	8	3.63E-11	3.97E-12	8	1.89E-11	3.67E-12	8	2.41E-11	3.80E-12	7	1.96E-11	5.13E-12
N-oleoyl alanine	Veh 1 hr	8	1.35E-11	1.41E-12	8	1.11E-11	1.20E-12	8	2.12E-11	2.17E-12	8	3.82E-11	1.64E-12	8	1.65E-11	1.39E-12	8	6.69E-12	8.67E-13
	Veh 3 hr	8	1.10E-11	1.06E-12	8	8.93E-12	9.05E-13	8	1.82E-11	1.94E-12	8	3.86E-11	2.01E-12	8	1.38E-11	1.29E-12	8	9.38E-12	1.27E-12
	CG 1 hr	7	1.21E-11	1.31E-12	8	7.96E-12	9.75E-13	8	1.70E-11	8.36E-13	8	4.24E-11	2.43E-12	8	1.46E-11	8.87E-13	8	8.88E-12	1.14E-12
	CG 3 hr	8	1.30E-11	9.63E-13	7	1.21E-11	1.03E-12	8	1.77E-11	1.07E-12	8	3.83E-11	2.77E-12	8	1.65E-11	7.32E-13	7	9.44E-12	1.47E-12
N-linoleoyl alanine	Veh 1 hr	8	BDL		8	BDL		8	PISSR		8	PISSR		8	PISSR		8	PISSR	
	Veh 3 hr	8	BDL		8	BDL		8	PISSR		8	PISSR		8	PISSR		8	PISSR	
	CG 1 hr	8	BDL		8	BDL		8	PISSR		8	PISSR		8	PISSR		8	PISSR	
	CG 3 hr	8	BDL		8	BDL		8	PISSR		8	PISSR		8	PISSR		7	PISSR	
N-arachidonoyl alanine	Veh 1 hr	7	7.09E-11	2.77E-12	8	8.85E-11	5.05E-12		N/A			N/A			N/A			N/A	
	Veh 3 hr	8	6.23E-11	3.94E-12	8	7.14E-11	9.59E-12		N/A			N/A			N/A			N/A	
	CG 1 hr	7	6.75E-11	2.56E-12	8	7.19E-11	6.32E-12		N/A			N/A			N/A			N/A	
	CG 3 hr	8	6.44E-11	3.74E-12	5	8.95E-11	1.99E-12		N/A			N/A			N/A			N/A	
N-docosahexaenoyl alanine	Veh 1 hr	8	4.51E-12	5.15E-13	8	BDL		8	PISSR		8	PISSR		8	PISSR		8	PISSR	
	Veh 3 hr	8	4.62E-12	4.49E-13	8	BDL		8	PISSR		8	PISSR		8	PISSR		8	PISSR	
	CG 1 hr	6	4.25E-12	4.40E-13	8	BDL		8	PISSR		8	PISSR		8	PISSR		8	PISSR	
	CG 3 hr	7	5.92E-12	5.27E-13	8	BDL		8	PISSR		8	PISSR		8	PISSR		7	PISSR	
***N*-acyl aspartic acid**																			
N-palmitoyl aspartic acid	Veh 1 hr	7	2.23E-11	1.40E-12	7	4.83E-11	2.18E-12		N/A			N/A			N/A			N/A	
	Veh 3 hr	7	3.09E-11	1.88E-12	8	5.85E-11	1.07E-11		N/A			N/A			N/A			N/A	
	CG 1 hr	7	2.62E-11	2.85E-12	7	4.82E-11	3.61E-12		N/A			N/A			N/A			N/A	
	CG 3 hr	7	2.81E-11	1.40E-12	7	5.87E-11	2.21E-12		N/A			N/A			N/A			N/A	
N-stearoyl aspartic acid	Veh 1 hr	8	PISSR		8	BDL			N/A			N/A			N/A			N/A	
	Veh 3 hr	8	PISSR		8	BDL			N/A			N/A			N/A			N/A	
	CG 1 hr	8	PISSR		8	BDL			N/A			N/A			N/A			N/A	
	CG 3 hr	8	PISSR		8	BDL			N/A			N/A			N/A			N/A	
N-oleoyl aspartic acid	Veh 1 hr	8	BDL		8	BDL			N/A			N/A			N/A			N/A	

	Veh 3 hr	8	BDL		8	BDL			N/A			N/A			N/A			N/A	
	CG 1 hr	8	BDL		8	BDL			N/A			N/A			N/A			N/A	
	CG 3 hr	8	BDL		8	BDL			N/A			N/A			N/A			N/A	
N-linoleoyl aspartic acid	Veh 1 hr	8	BDL		8	BDL			N/A			N/A			N/A			N/A	
	Veh 3 hr	8	BDL		8	BDL			N/A			N/A			N/A			N/A	
	CG 1 hr	8	BDL		8	BDL			N/A			N/A			N/A			N/A	
	CG 3 hr	8	BDL		8	BDL			N/A			N/A			N/A			N/A	
***N*-acyl dopamine**																			
N-arachidonoyl dopamine	Veh 1 hr	8	8.53E-12	1.72E-12	8	BDL			N/A			N/A			N/A			N/A	
	Veh 3 hr	8	6.92E-12	1.59E-12	8	BDL			N/A			N/A			N/A			N/A	
	CG 1 hr	7	7.36E-12	1.96E-12	8	BDL			N/A			N/A			N/A			N/A	
	CG 3 hr	8	1.00E-11	2.23E-12	8	BDL			N/A			N/A			N/A			N/A	
***N*-acyl ethanolamine**																			
N-palmitoyl ethanolamine	Veh 1 hr	7	1.72E-10	9.70E-12	8	9.61E-11	1.16E-11	8	6.74E-11	6.34E-12	8	1.25E-10	1.93E-11	8	1.55E-10	2.09E-11	8	1.86E-10	1.84E-11
	Veh 3 hr	8	1.52E-10	9.04E-12	8	7.20E-11	1.01E-11	8	5.20E-11	2.76E-12	8	7.60E-11	7.27E-12	8	1.17E-10	7.95E-12	8	1.67E-10	9.78E-12
	CG 1 hr	7	1.61E-10	1.41E-11	8	8.11E-11	1.02E-11	8	6.14E-11	1.73E-12	8	1.05E-10	1.43E-11	8	1.44E-10	9.36E-12	8	1.84E-10	1.97E-11
	CG 3 hr	8	2.08E-10	1.39E-11	6	1.07E-10	8.55E-12	8	8.45E-11	7.93E-12	8	1.09E-10	7.53E-12	8	1.53E-10	8.29E-12	7	2.32E-10	1.87E-11
N-stearoyl ethanolamine	Veh 1 hr	8	7.02E-10	8.95E-11	8	3.52E-10	4.84E-11	8	3.03E-10	3.33E-11	8	6.66E-10	1.17E-10	8	6.39E-10	9.79E-11	8	6.39E-10	8.67E-11
	Veh 3 hr	8	6.25E-10	6.25E-11	8	2.92E-10	5.74E-11	8	2.88E-10	5.14E-11	8	4.33E-10	7.33E-11	8	4.94E-10	6.52E-11	8	6.68E-10	6.89E-11
	CG 1 hr	7	6.37E-10	8.50E-11	8	3.19E-10	3.13E-11	8	3.19E-10	2.58E-11	8	5.50E-10	4.86E-11	8	6.32E-10	4.68E-11	8	7.53E-10	9.64E-11
	CG 3 hr	8	8.45E-10	9.75E-11	7	3.83E-10	3.74E-11	8	3.90E-10	6.19E-11	8	5.27E-10	7.64E-11	8	6.65E-10	4.42E-11	7	8.47E-10	6.91E-11
N-oleoyl ethanolamine	Veh 1 hr	7	3.78E-10	2.25E-11	8	2.36E-10	2.17E-11	8	2.31E-10	1.93E-11	8	2.84E-10	3.83E-11	8	3.55E-10	4.18E-11	8	4.41E-10	3.97E-11
	Veh 3 hr	8	3.32E-10	2.02E-11	8	1.89E-10	2.85E-11	8	1.62E-10	1.07E-11	8	1.73E-10	1.67E-11	8	2.58E-10	1.80E-11	8	3.87E-10	2.84E-11
	CG 1 hr	7	3.61E-10	2.58E-11	8	2.09E-10	1.92E-11	8	2.00E-10	8.47E-12	8	2.25E-10	2.47E-11	8	3.20E-10	1.75E-11	8	4.09E-10	4.84E-11
	CG 3 hr	8	4.63E-10	2.86E-11	6	2.67E-10	1.40E-11	8	2.72E-10	2.66E-11	8	2.37E-10	1.67E-11	8	3.49E-10	1.78E-11	7	5.13E-10	3.13E-11
N-linoleoyl ethanolamine	Veh 1 hr	7	1.59E-10	1.04E-11	7	6.86E-11	2.89E-12	8	3.42E-11	1.34E-12	8	2.42E-11	2.94E-12	8	3.53E-11	1.98E-12	8	2.09E-11	1.71E-12
	Veh 3 hr	8	1.49E-10	7.86E-12	8	5.91E-11	8.33E-12	8	2.74E-11	1.77E-12	8	1.61E-11	2.20E-12	8	2.55E-11	1.55E-12	8	1.80E-11	1.28E-12
	CG 1 hr	7	1.55E-10	1.13E-11	8	7.25E-11	5.18E-12	8	3.40E-11	1.61E-12	8	2.05E-11	2.57E-12	8	3.18E-11	1.97E-12	8	1.99E-11	2.22E-12
	CG 3 hr	8	2.26E-10	1.71E-11	7	9.14E-11	3.15E-12	8	4.44E-11	2.31E-12	8	2.29E-11	1.88E-12	8	3.54E-11	2.34E-12	7	2.37E-11	1.90E-12
N-arachidonoyl ethanolamine	Veh 1 hr	7	7.30E-11	5.73E-12	8	7.59E-11	7.66E-12	8	1.93E-11	1.40E-12	8	1.76E-11	2.68E-12	8	2.66E-11	2.53E-12	8	1.80E-11	2.01E-12
	Veh 3 hr	8	6.56E-11	4.46E-12	8	6.00E-11	9.11E-12	8	1.43E-11	9.71E-13	8	1.11E-11	1.68E-12	8	1.81E-11	1.38E-12	8	1.54E-11	1.16E-12
	CG 1 hr	7	6.90E-11	4.22E-12	8	7.25E-11	6.33E-12	8	1.83E-11	4.74E-13	8	1.46E-11	2.39E-12	8	2.17E-11	1.48E-12	8	1.64E-11	2.15E-12
	CG 3 hr	8	9.46E-11	6.89E-12	6	8.54E-11	2.87E-12	8	2.60E-11	1.59E-12	8	1.65E-11	1.36E-12	8	2.38E-11	1.46E-12	7	2.10E-11	1.61E-12
N-docosahexaenoyl ethanolamine	Veh 1 hr	8	1.00E-10	8.11E-12	7	4.93E-11	1.74E-12	8	3.57E-11	2.06E-12	8	3.23E-11	4.24E-12	8	6.04E-11	5.27E-12	8	5.70E-11	4.97E-12
	Veh 3 hr	8	7.91E-11	4.60E-12	8	4.07E-11	5.64E-12	8	2.77E-11	2.15E-12	8	2.13E-11	2.46E-12	8	4.91E-11	4.23E-12	8	4.96E-11	3.71E-12
	CG 1 hr	7	8.58E-11	5.27E-12	8	4.53E-11	4.27E-12	8	3.45E-11	1.39E-12	8	2.75E-11	3.10E-12	8	5.38E-11	2.53E-12	8	5.09E-11	5.09E-12
	CG 3 hr	8	1.176E-10	7.91E-12	7	5.97E-11	2.95E-12	8	4.85E-11	2.19E-12	8	3.25E-11	2.12E-12	8	5.79E-11	2.79E-12	7	6.03E-11	3.09E-12
***N*-acyl GABA**																			
N-palmitoyl GABA	Veh 1 hr	8	6.22E-11	9.02E-12	8	3.79E-11	8.76E-12	8	5.35E-11	3.60E-12	8	6.44E-11	6.63E-12	8	6.26E-11	5.94E-12	8	3.49E-11	2.06E-12

	Veh 3 hr	7	4.95E-11	2.88E-12	8	3.21E-11	8.41E-12	8	4.39E-11	3.58E-12	8	6.23E-11	6.52E-12	8	5.27E-11	3.92E-12	8	3.44E-11	2.22E-12
	CG 1 hr	6	4.53E-11	2.47E-12	8	3.67E-11	7.02E-12	8	4.48E-11	2.35E-12	8	6.41E-11	2.46E-12	8	5.55E-11	2.42E-12	8	3.13E-11	2.13E-12
	CG 3 hr	8	5.43E-11	4.93E-12	6	3.23E-11	7.96E-12	8	6.29E-11	7.63E-12	8	5.82E-11	5.00E-12	8	5.32E-11	2.86E-12	7	4.04E-11	3.35E-12
N-stearoyl GABA	Veh 1 hr	8	1.01E-11	1.12E-12	8	4.43E-12	3.93E-13	8	1.11E-11	6.37E-13	8	2.57E-11	6.48E-12	8	9.53E-12	1.08E-12	8	9.47E-12	9.11E-13
	Veh 3 hr	8	9.26E-12	5.34E-13	8	3.64E-12	6.23E-13	8	9.44E-12	7.60E-13	8	2.38E-11	4.57E-12	8	7.50E-12	5.17E-13	8	9.41E-12	1.14E-12
	CG 1 hr	5	8.50E-12	6.17E-13	7	3.35E-12	1.81E-13	8	1.04E-11	6.39E-13	8	2.58E-11	5.17E-12	8	9.11E-12	7.17E-13	8	8.36E-12	1.34E-12
	CG 3 hr	8	8.88E-12	8.05E-13	7	3.98E-12	5.56E-13	8	1.48E-11	1.98E-12	8	2.35E-11	4.12E-12	8	8.24E-12	4.92E-13	7	1.09E-11	9.01E-13
N-oleoyl GABA	Veh 1 hr	7	1.30E-11	1.30E-11	8	1.10E-11	2.73E-12	8	1.24E-11	8.63E-13	8	2.97E-11	7.51E-12	8	1.34E-11	1.62E-12	8	1.06E-11	9.56E-13
	Veh 3 hr	8	1.17E-11	1.17E-11	8	7.17E-12	1.72E-12	8	9.29E-12	7.60E-13	8	2.55E-11	5.59E-12	8	1.03E-11	7.98E-13	8	1.06E-11	1.04E-12
	CG 1 hr	6	1.10E-11	1.10E-11	8	8.35E-12	1.03E-12	8	1.09E-11	7.15E-13	8	2.83E-11	4.62E-12	8	1.18E-11	6.49E-13	8	1.01E-11	1.39E-12
	CG 3 hr	8	1.32E-11	1.32E-11	7	1.05E-11	2.74E-12	8	1.67E-11	2.12E-12	8	2.65E-11	4.53E-12	8	1.21E-11	5.44E-13	7	1.21E-11	7.18E-13
N-linoleoyl GABA	Veh 1 hr	7	1.63E-12	2.10E-13	8	BDL		8	1.24E-11	8.63E-13	8	1.58E-12	2.49E-13	8	1.30E-12	2.68E-13	8	6.57E-13	5.41E-14
	Veh 3 hr	8	1.08E-12	1.23E-13	8	BDL		8	9.29E-12	7.60E-13	8	1.28E-12	2.55E-13	8	1.00E-12	1.15E-13	8	6.43E-13	4.37E-14
	CG 1 hr	7	1.86E-12	2.78E-13	8	BDL		8	1.09E-11	7.15E-13	8	1.48E-12	1.29E-13	8	1.33E-12	2.22E-13	8	6.29E-13	1.01E-13
	CG 3 hr	8	1.57E-12	2.18E-13	8	BDL		8	1.67E-11	2.12E-12	8	1.67E-12	2.56E-13	8	1.04E-12	1.41E-13	7	7.11E-13	7.06E-14
N-arachidonoyl GABA	Veh 1 hr	7	4.64E-11	2.74E-12	8	PISSR		8	3.22E-11	3.15E-12	8	2.70E-11	3.22E-12	8	2.49E-11	2.82E-12	8	8.14E-12	7.49E-13
	Veh 3 hr	8	4.00E-11	2.61E-12	8	PISSR		8	2.20E-11	1.78E-12	8	2.17E-11	1.97E-12	8	1.77E-11	1.65E-12	8	7.12E-12	5.74E-13
	CG 1 hr	7	4.28E-11	2.48E-12	8	PISSR		8	2.45E-11	9.48E-13	8	2.54E-11	9.35E-13	8	2.00E-11	1.50E-12	8	6.15E-12	7.35E-13
	CG 3 hr	8	4.72E-11	3.78E-12	8	PISSR		8	3.69E-11	4.02E-12	8	2.24E-11	1.99E-12	8	2.08E-11	1.37E-12	7	8.46E-12	6.20E-13
N-docosahexaenoyl GABA	Veh 1 hr		N/A		8	BDL		8	1.88E-13	6.26E-14	8	2.12E-11	4.83E-12	8	9.23E-12	1.16E-12	8	1.08E-13	1.37E-14
	Veh 3 hr		N/A		8	BDL		8	1.64E-13	6.12E-15	8	2.05E-11	3.85E-12	8	9.90E-12	1.30E-12	8	1.05E-13	2.33E-14
	CG 1 hr		N/A		8	BDL		8	1.63E-13	2.93E-14	8	2.18E-11	2.56E-12	8	1.08E-11	1.22E-12	8	1.11E-13	2.09E-14
	CG 3 hr		N/A		8	BDL		8	2.46E-13	3.79E-14	8	1.92E-11	3.48E-12	8	1.19E-11	1.05E-12	7	1.40E-13	1.28E-14
***N*-acyl glycine**																			
N-palmitoyl glycine	Veh 1 hr	8	1.84E-11	6.50E-12	7	6.17E-11	5.04E-12	8	3.77E-11	3.03E-12	8	4.55E-11	6.59E-12	8	7.09E-11	3.01E-12	8	1.42E-10	2.24E-11
	Veh 3 hr	7	9.73E-12	3.68E-12	8	6.32E-11	5.68E-12	8	3.82E-11	6.75E-12	8	4.55E-11	5.73E-12	8	6.73E-11	3.40E-12	8	1.67E-10	1.48E-11
	CG 1 hr	6	1.37E-11	5.59E-12	8	6.26E-11	4.24E-12	8	4.09E-11	6.56E-12	8	4.63E-11	2.59E-12	8	6.71E-11	3.31E-12	8	1.43E-10	1.62E-11
	CG 3 hr	8	2.21E-11	7.82E-12	7	7.81E-11	6.88E-12	8	4.63E-11	7.46E-12	8	4.48E-11	3.46E-12	8	7.53E-11	5.91E-12	7	1.37E-10	2.22E-11
N-stearoyl glycine	Veh 1 hr	8	8.07E-11	7.30E-12	8	8.75E-11	1.35E-11	8	1.91E-11	2.10E-12	8	2.63E-11	4.84E-12	8	9.50E-11	7.97E-12	8	8.50E-11	1.88E-11
	Veh 3 hr	8	8.07E-11	5.99E-12	8	7.88E-11	1.42E-11	8	1.81E-11	1.21E-12	8	2.16E-11	4.31E-12	8	8.70E-11	5.07E-12	8	1.06E-10	1.23E-11
	CG 1 hr	7	7.87E-11	5.67E-12	8	9.28E-11	6.01E-12	8	1.94E-11	2.11E-12	8	2.07E-11	5.37E-12	8	8.85E-11	6.12E-12	8	8.57E-11	1.79E-11
	CG 3 hr	8	8.24E-11	5.29E-12	7	8.59E-11	1.60E-11	8	2.70E-11	4.85E-12	8	2.02E-11	3.42E-12	8	9.76E-11	6.21E-12	7	9.11E-11	2.72E-11
N-oleoyl glycine	Veh 1 hr	8	6.90E-11	7.24E-12	7	4.99E-11	6.34E-12	8	7.13E-11	3.08E-12	8	9.23E-11	1.35E-11	8	4.91E-11	6.09E-12	8	1.54E-10	1.91E-11
	Veh 3 hr	8	4.61E-11	2.94E-12	8	3.46E-11	5.70E-12	8	6.03E-11	3.65E-12	8	7.90E-11	1.41E-11	8	4.19E-11	2.17E-12	8	1.68E-10	1.64E-11
	CG 1 hr	7	4.94E-11	4.32E-12	7	3.61E-11	2.46E-12	8	6.66E-11	3.73E-12	8	8.12E-11	1.03E-11	8	4.84E-11	3.55E-12	8	1.37E-10	2.04E-11
	CG 3 hr	7	5.74E-11	3.86E-12	7	5.68E-11	7.58E-12	8	1.03E-10	9.85E-12	8	8.64E-11	9.23E-12	8	5.14E-11	3.50E-12	7	1.51E-10	3.14E-11
N-linoleoyl glycine	Veh 1 hr	8	1.02E-11	1.29E-12	8	1.00E-11	2.49E-12	8	1.26E-10	1.52E-11	8	9.12E-11	2.04E-11	8	6.41E-12	1.30E-12	8	1.86E-11	4.30E-13
	Veh 3 hr	8	6.69E-12	5.29E-13	7	4.64E-12	6.84E-13	8	1.01E-10	1.73E-11	8	8.72E-11	1.25E-11	8	5.50E-12	8.16E-13	8	2.02E-11	6.35E-13
	CG 1 hr	7	8.40E-12	1.06E-12	8	6.09E-12	8.44E-13	8	1.13E-10	1.79E-11	8	1.02E-10	1.41E-11	8	6.64E-12	2.02E-12	8	1.69E-11	1.18E-12

	CG 3 hr	7	9.27E-12	1.33E-12	7	8.10E-12	1.76E-12	8	1.60E-10	1.94E-11	8	9.57E-11	1.24E-11	8	6.59E-12	1.18E-12	7	1.88E-11	8.37E-13
N-arachidonoyl glycine	Veh 1 hr	7	2.07E-10	8.84E-12	7	2.16E-10	2.13E-11	8	5.35E-11	5.30E-12	8	4.15E-11	8.84E-12	8	8.02E-11	1.95E-11	8	5.81E-11	2.20E-12
	Veh 3 hr	8	1.72E-10	1.06E-11	8	1.84E-10	1.93E-11	8	4.30E-11	5.83E-12	8	3.90E-11	4.66E-12	8	9.08E-11	1.20E-11	8	6.16E-11	1.57E-12
	CG 1 hr	7	1.77E-10	8.54E-12	7	2.21E-10	7.06E-12	8	5.08E-11	6.72E-12	8	4.60E-11	6.27E-12	8	9.06E-11	2.16E-11	8	5.53E-11	2.00E-12
	CG 3 hr	8	2.05E-10	2.02E-11	7	2.62E-10	2.28E-11	8	6.91E-11	6.88E-12	8	4.23E-11	3.43E-12	8	1.07E-10	2.24E-11	7	5.96E-11	1.44E-12
N-docosahexaenoyl glycine	Veh 1 hr	8	1.52E-11	1.12E-12	7	6.97E-12	9.20E-13	8	1.02E-11	8.74E-13	8	1.16E-11	2.45E-12	8	4.88E-12	1.11E-12	8	2.07E-12	6.86E-14
	Veh 3 hr	8	1.16E-11	9.47E-13	8	5.88E-12	8.31E-13	8	9.17E-12	1.05E-12	8	1.19E-11	2.36E-12	8	7.24E-12	9.24E-13	8	2.17E-12	3.92E-13
	CG 1 hr	7	1.32E-11	1.30E-12	8	9.06E-12	9.46E-13	8	1.00E-11	1.29E-12	8	1.25E-11	1.53E-12	8	6.57E-12	1.72E-12	8	1.71E-12	2.22E-13
	CG 3 hr	8	1.76E-11	1.55E-12	7	1.02E-11	1.74E-12	8	1.45E-11	1.49E-12	8	1.17E-11	1.45E-12	8	7.43E-12	1.43E-12	7	1.59E-12	1.28E-13
***N*-acyl leucine**																			
N-palmitoyl leucine	Veh 1 hr	8	1.07E-11	5.90E-13	8	6.45E-12	4.51E-13	8	8.20E-12	2.72E-12		N/A		8	1.19E-11	1.05E-12	8	1.43E-11	5.04E-12
	Veh 3 hr	8	1.16E-11	6.29E-13	8	7.78E-12	7.38E-13	8	8.86E-12	4.65E-12		N/A		8	1.08E-11	1.36E-12	8	1.11E-11	2.70E-12
	CG 1 hr	7	1.14E-11	1.03E-12	8	6.52E-12	6.07E-13	8	4.50E-12	4.55E-13		N/A		8	1.01E-11	7.34E-13	8	1.32E-11	4.34E-12
	CG 3 hr	8	1.25E-11	1.05E-12	7	8.26E-12	7.24E-13	8	5.54E-12	6.08E-13		N/A		8	1.28E-11	2.23E-12	7	1.59E-11	8.02E-12
N-stearoyl leucine	Veh 1 hr	8	8.89E-12	4.18E-13	7	7.71E-12	3.25E-13	8	1.21E-11	1.19E-12		N/A		8	1.14E-11	9.57E-13	8	1.95E-11	3.83E-12
	Veh 3 hr	8	8.71E-12	5.95E-13	8	8.75E-12	6.08E-13	8	1.24E-11	2.11E-12		N/A		8	9.46E-12	4.93E-13	8	1.86E-11	2.60E-12
	CG 1 hr	6	8.82E-12	1.84E-13	8	7.67E-12	3.72E-13	8	1.11E-11	3.92E-13		N/A		8	1.03E-11	5.28E-13	8	1.86E-11	2.57E-12
	CG 3 hr	7	9.16E-12	3.59E-13	7	8.03E-12	2.71E-13	8	1.28E-11	1.52E-12		N/A		8	1.12E-11	1.02E-12	7	2.13E-11	4.90E-12
N-oleoyl leucine	Veh 1 hr	7	2.39E-12	2.95E-13	8	2.33E-12	2.20E-13	8	3.50E-12	5.79E-13		N/A		8	3.57E-12	3.72E-13	8	1.28E-11	5.56E-12
	Veh 3 hr	8	2.77E-12	2.83E-13	8	2.48E-12	2.65E-13	8	3.80E-12	1.06E-12		N/A		8	3.16E-12	7.62E-14	8	6.85E-12	1.65E-12
	CG 1 hr	7	2.49E-12	2.84E-13	8	1.96E-12	1.77E-13	8	2.66E-12	1.69E-13		N/A		8	3.93E-12	2.29E-13	8	5.31E-12	4.30E-13
	CG 3 hr	7	2.75E-12	2.56E-13	7	2.32E-12	1.23E-13	8	3.46E-12	3.74E-13		N/A		8	4.04E-12	2.44E-13	7	7.90E-12	2.35E-12
N-linoleoyl leucine	Veh 1 hr	8	4.77E-13	1.17E-13	8	PISSR		8	6.30E-13	1.04E-13		N/A		8	3.96E-13	8.03E-14	8	9.51E-13	1.72E-13
	Veh 3 hr	8	3.83E-13	6.14E-14	8	PISSR		8	5.40E-13	6.76E-14		N/A		8	2.66E-13	3.45E-14	8	9.06E-13	9.31E-14
	CG 1 hr	7	3.88E-13	5.45E-14	8	PISSR		8	5.90E-13	7.42E-14		N/A		8	3.59E-13	6.25E-14	8	7.74E-13	5.24E-14
	CG 3 hr	8	5.94E-13	1.44E-13	8	PISSR		8	6.87E-13	5.77E-14		N/A		8	4.40E-13	7.28E-14	7	1.02E-12	9.03E-14
N-docosahexaenoyl leucine	Veh 1 hr	8	3.66E-12	2.84E-13	8	1.86E-12	3.11E-13	8	3.65E-12	1.92E-13		N/A		8	2.99E-12	2.18E-13	8	2.83E-12	3.67E-13
	Veh 3 hr	8	4.26E-12	3.94E-13	8	1.71E-12	4.22E-13	8	3.51E-12	2.60E-13		N/A		8	2.25E-12	2.12E-13	8	2.84E-12	3.97E-13
	CG 1 hr	6	3.49E-12	1.47E-13	6	1.86E-12	2.13E-13	8	3.41E-12	2.25E-13		N/A		8	2.67E-12	2.73E-13	8	2.60E-12	2.50E-13
	CG 3 hr	8	3.80E-12	2.47E-13	8	1.57E-12	1.17E-13	8	4.17E-12	5.12E-13		N/A		8	2.89E-12	2.33E-13	7	2.80E-12	1.41E-13
***N*-acyl methionine**																			
N-palmitoyl methionine	Veh 1 hr	8	3.30E-11	4.17E-12	7	2.81E-11	1.76E-12		N/A		8	4.41E-12	5.73E-13	8	1.80E-11	1.28E-12	8	6.93E-12	9.22E-13
	Veh 3 hr	8	3.42E-11	3.55E-12	8	3.83E-11	4.15E-12		N/A		8	4.72E-12	7.09E-13	8	1.58E-11	1.27E-12	8	7.42E-12	1.54E-12
	CG 1 hr	6	2.81E-11	1.49E-12	8	2.58E-11	3.74E-12		N/A		8	6.08E-12	1.07E-12	8	1.57E-11	1.63E-12	8	7.41E-12	1.48E-12
	CG 3 hr	7	3.01E-11	2.30E-12	7	2.92E-11	5.64E-12		N/A		8	4.79E-12	9.64E-13	8	1.72E-11	1.14E-12	7	8.18E-12	1.26E-12
N-stearoyl methionine	Veh 1 hr	6	4.66E-12	1.65E-13	8	PISSR			N/A		8	3.96E-12	4.41E-13	8	5.13E-12	6.71E-13	8	6.50E-12	9.40E-13
	Veh 3 hr	8	5.04E-12	2.49E-13	8	PISSR			N/A		8	3.66E-12	3.22E-13	8	5.52E-12	2.05E-13	8	6.85E-12	7.02E-13
	CG 1 hr	7	4.73E-12	3.28E-13	8	PISSR			N/A		8	4.49E-12	1.96E-13	8	5.51E-12	3.06E-13	8	6.93E-12	8.29E-13
	CG 3 hr	8	4.52E-12	1.58E-13	8	PISSR			N/A		8	3.63E-12	3.30E-13	8	5.01E-12	3.15E-13	7	7.76E-12	5.34E-13

N-oleoyl methionine	Veh 1 hr	8	3.88E-12	4.06E-13	8	PISSR			N/A		8	2.83E-12	4.26E-13	8	5.63E-12	6.23E-13	8	4.89E-12	8.76E-13
	Veh 3 hr	8	4.73E-12	2.72E-13	8	PISSR			N/A		8	2.74E-12	3.62E-13	8	5.97E-12	4.83E-13	8	6.68E-12	9.25E-13
	CG 1 hr	7	4.15E-12	5.72E-13	8	PISSR			N/A		8	2.99E-12	3.65E-13	8	5.88E-12	5.35E-13	8	6.33E-12	7.82E-13
	CG 3 hr	8	4.13E-12	4.91E-13	8	PISSR			N/A		8	2.58E-12	3.36E-13	8	5.69E-12	2.25E-13	7	6.60E-12	5.78E-13
N-linoleoyl methionine	Veh 1 hr	8	5.29E-12	4.18E-13	8	PISSR			N/A					8	3.92E-12	5.18E-13	8	2.01E-12	1.45E-13
	Veh 3 hr	8	5.86E-12	4.50E-13	8	PISSR			N/A					8	3.57E-12	2.56E-13	8	2.05E-12	1.13E-13
	CG 1 hr	7	5.24E-12	8.13E-13	8	PISSR			N/A					8	4.62E-12	3.44E-13	8	1.81E-12	8.54E-14
	CG 3 hr	8	7.41E-12	4.37E-13	8	PISSR			N/A					8	4.50E-12	3.77E-13	7	1.86E-12	1.55E-13
N-arachidonoyl methionine	Veh 1 hr	8	BDL		8	PISSR		8	N/A		8	PISSR		8	PISSR		8	PISSR	
	Veh 3 hr	8	BDL		8	PISSR		8	N/A		8	PISSR		8	PISSR		8	PISSR	
	CG 1 hr	8	BDL		8	PISSR		8	N/A		8	PISSR		8	PISSR		8	PISSR	
	CG 3 hr	8	BDL		8	PISSR		8	N/A		8	PISSR		8	PISSR		7	PISSR	
N-docosahexaenoyl methionine	Veh 1 hr	8	BDL		8	BDL		8	N/A		8	PISSR		8	PISSR		8	PISSR	
	Veh 3 hr	8	BDL		8	BDL		8	N/A		8	PISSR		8	PISSR		8	PISSR	
	CG 1 hr	8	BDL		8	BDL		8	N/A		8	PISSR		8	PISSR		8	PISSR	
	CG 3 hr	8	BDL		8	BDL		8	N/A		8	PISSR		8	PISSR		7	PISSR	
***N*-acyl phenylalanine**																			
N-palmitoyl phenylalanine	Veh 1 hr	7	9.00E-12	3.79E-13	8	3.24E-12	5.02E-13		N/A			N/A			N/A			N/A	
	Veh 3 hr	8	1.18E-11	7.26E-13	7	7.32E-12	7.07E-13		N/A			N/A			N/A			N/A	
	CG 1 hr	6	1.02E-11	8.13E-13	8	6.30E-12	9.21E-13		N/A			N/A			N/A			N/A	
	CG 3 hr	8	1.25E-11	2.42E-13	7	7.19E-12	1.39E-12		N/A			N/A			N/A			N/A	
N-stearoyl phenylalanine	Veh 1 hr	8	4.59E-12	2.17E-13	8	PISSR			N/A			N/A			N/A			N/A	
	Veh 3 hr	8	4.56E-12	2.76E-13	8	PISSR			N/A			N/A			N/A			N/A	
	CG 1 hr	5	4.10E-12	2.59E-13	8	PISSR			N/A			N/A			N/A			N/A	
	CG 3 hr	7	5.09E-12	1.75E-13	8	PISSR			N/A			N/A			N/A			N/A	
N-linoleoyl phenylalanine	Veh 1 hr		N/A		8	BDL			N/A			N/A			N/A			N/A	
	Veh 3 hr		N/A		8	BDL			N/A			N/A			N/A			N/A	
	CG 1 hr		N/A		8	BDL			N/A			N/A			N/A			N/A	
	CG 3 hr		N/A		8	BDL			N/A			N/A			N/A			N/A	
N-arachidonoyl phenylalanine	Veh 1 hr	8	BDL		8	BDL			N/A			N/A			N/A			N/A	
	Veh 3 hr	8	BDL		8	BDL			N/A			N/A			N/A			N/A	
	CG 1 hr	8	BDL		8	BDL			N/A			N/A			N/A			N/A	
	CG 3 hr	8	BDL		8	BDL			N/A			N/A			N/A			N/A	
N-docosahexaenoyl phenylalanine	Veh 1 hr	8	BDL		8	BDL			N/A			N/A			N/A			N/A	
	Veh 3 hr	8	BDL		8	BDL			N/A			N/A			N/A			N/A	
	CG 1 hr	8	BDL		8	BDL			N/A			N/A			N/A			N/A	
	CG 3 hr	8	BDL		8	BDL			N/A			N/A			N/A			N/A	
***N*-acyl proline**																			
N-palmitoyl proline	Veh 1 hr	8	BDL		8	BDL			N/A		8	1.49E-12	1.21E-13	8	8.48E-12	5.24E-13	8	4.47E-12	3.17E-12

	Veh 3 hr	8	BDL		8	BDL			N/A		8	1.66E-12	1.41E-13	8	7.33E-12	7.76E-13	8	1.60E-12	2.12E-13
	CG 1 hr	8	BDL		8	BDL			N/A		8	1.56E-12	2.34E-13	8	7.26E-12	5.22E-13	8	1.42E-12	2.63E-13
	CG 3 hr	8	BDL		8	BDL			N/A		8	1.69E-12	1.34E-13	8	8.82E-12	1.15E-12	7	2.23E-12	6.95E-13
N-stearoyl proline	Veh 1 hr	8	BDL		8	BDL			N/A		8	1.10E-12	1.94E-13	8	7.95E-13	8.14E-14	8	3.41E-12	2.65E-12
	Veh 3 hr	8	BDL		8	BDL			N/A		8	7.29E-13	8.40E-14	8	7.14E-13	1.28E-13	8	7.96E-13	1.72E-13
	CG 1 hr	8	BDL		8	BDL			N/A		8	9.22E-13	1.51E-13	8	5.01E-13	3.68E-14	8	9.26E-13	1.12E-13
	CG 3 hr	8	BDL		8	BDL			N/A		8	1.09E-12	1.60E-13	8	9.73E-13	2.14E-13	7	9.06E-13	1.40E-13
N-oleoyl proline	Veh 1 hr	8	BDL		8	BDL		8	PISSR		8	PISSR		8	PISSR		8	PISSR	
	Veh 3 hr	8	BDL		8	BDL		8	PISSR		8	PISSR		8	PISSR		8	PISSR	
	CG 1 hr	8	BDL		8	BDL		8	PISSR		8	PISSR		8	PISSR		8	PISSR	
	CG 3 hr	8	BDL		8	BDL		8	PISSR		8	PISSR		8	PISSR		7	PISSR	
N-linoleoyl proline	Veh 1 hr	8	BDL		8	BDL		8	BDL		8	BDL		8	BDL		8	BDL	
	Veh 3 hr	8	BDL		8	BDL		8	BDL		8	BDL		8	BDL		8	BDL	
	CG 1 hr	8	BDL		8	BDL		8	BDL		8	BDL		8	BDL		8	BDL	
	CG 3 hr	8	BDL		8	BDL		8	BDL		8	BDL		8	BDL		7	BDL	
N-arachidonoyl proline	Veh 1 hr	8	BDL		8	BDL		8	BDL		8	BDL		8	BDL		8	BDL	
	Veh 3 hr	8	BDL		8	BDL		8	BDL		8	BDL		8	BDL		8	BDL	
	CG 1 hr	8	BDL		8	BDL		8	BDL		8	BDL		8	BDL		8	BDL	
	CG 3 hr	8	BDL		8	BDL		8	BDL		8	BDL		8	BDL		8	BDL	
N-docosahexaenoyl proline	Veh 1 hr	8	BDL		8	BDL		8	BDL		8	BDL		8	BDL		7	BDL	
	Veh 3 hr	8	BDL		8	BDL		8	BDL		8	BDL		8	BDL		8	BDL	
	CG 1 hr	8	BDL		8	BDL		8	BDL		8	BDL		8	BDL		8	BDL	
	CG 3 hr	8	BDL		8	BDL		8	BDL		8	BDL		8	BDL		7	BDL	
***N*-acyl serine**																			
N-palmitoyl serine	Veh 1 hr	7	9.90E-11	4.05E-12	8	5.77E-11	5.07E-12	8	1.81E-11	3.12E-12	8	2.89E-11	6.06E-12	8	7.90E-11	4.17E-12	8	1.77E-10	2.68E-11
	Veh 3 hr	8	1.13E-10	4.81E-12	8	6.57E-11	4.69E-12	8	1.61E-11	3.06E-12	8	3.35E-11	4.59E-12	8	6.20E-11	3.02E-12	8	2.45E-10	2.48E-11
	CG 1 hr	7	1.07E-10	5.62E-12	8	6.87E-11	4.00E-12	8	1.82E-11	3.34E-12	8	3.92E-11	6.06E-12	8	6.85E-11	6.01E-12	8	2.33E-10	2.62E-11
	CG 3 hr	8	1.04E-10	4.41E-12	7	5.83E-11	6.33E-12	8	1.66E-11	1.89E-12	8	3.27E-11	4.65E-12	8	7.21E-11	3.27E-12	7	2.43E-10	3.05E-11
N-stearoyl serine	Veh 1 hr	8	1.15E-10	3.93E-12	7	1.10E-10	4.59E-12	8	1.94E-11	7.46E-13	8	2.59E-11	2.34E-12	8	6.27E-11	4.64E-12	8	7.55E-10	1.13E-10
	Veh 3 hr	8	1.24E-10	6.28E-12	8	1.32E-10	7.37E-12	8	2.05E-11	7.62E-13	8	2.54E-11	2.00E-12	8	5.26E-11	2.93E-12	8	6.92E-10	1.17E-10
	CG 1 hr	6	.03E-101	5.79E-12	8	1.37E-10	9.80E-12	8	1.92E-11	6.15E-13	8	2.55E-11	1.78E-12	8	5.68E-11	2.33E-12	8	7.03E-10	1.27E-10
	CG 3 hr	7	1.26E-10	2.86E-12	7	1.04E-10	1.98E-11	8	2.27E-11	2.39E-12	8	2.49E-11	1.32E-12	8	5.62E-11	2.51E-12	7	9.00E-10	1.08E-10
N-oleoyl serine	Veh 1 hr	8	1.40E-10	1.28E-11	8	2.01E-10	9.93E-12	8	2.33E-11	1.46E-12	8	2.59E-11	2.73E-12	8	6.50E-11	6.96E-12	8	1.66E-10	1.91E-11
	Veh 3 hr	8	1.48E-10	1.24E-11	7	2.09E-10	1.74E-11	8	2.37E-11	1.41E-12	8	2.75E-11	1.64E-12	8	6.32E-11	4.06E-12	8	2.26E-10	1.74E-11
	CG 1 hr	6	1.26E-10	1.27E-11	7	2.12E-10	7.65E-12	8	2.37E-11	2.11E-12	8	2.98E-11	1.45E-12	8	6.65E-11	2.90E-12	8	2.27E-10	2.05E-11
	CG 3 hr	8	1.45E-10	7.33E-12	7	2.38E-10	1.28E-11	8	2.71E-11	2.51E-12	8	2.66E-11	1.76E-12	8	7.07E-11	3.24E-12	7	2.26E-10	2.33E-11
N-linoleoyl serine	Veh 1 hr	8	5.17E-11	5.72E-12	8	1.96E-11	2.63E-12	8	7.21E-12	6.54E-13	7	8.18E-12	7.88E-13	8	3.77E-11	4.86E-12	8	5.84E-12	7.00E-13
	Veh 3 hr	8	5.39E-11	6.98E-12	7	2.13E-11	2.97E-12	8	7.22E-12	4.88E-13	8	8.56E-12	6.42E-13	7	1.96E-11	3.35E-12	8	7.55E-12	7.44E-13
	CG 1 hr	7	5.34E-11	6.05E-12	8	2.41E-11	3.08E-12	8	7.20E-12	7.90E-13	8	8.42E-12	7.10E-13	7	2.54E-11	5.49E-12	8	6.65E-12	4.75E-13

	CG 3 hr	8	5.24E-11	4.35E-12	7	3.04E-11	3.89E-12	8	7.90E-12	7.97E-13	8	7.98E-12	6.43E-13	7	3.16E-11	5.56E-12	7	7.68E-12	8.14E-13
N-arachidonoyl serine	Veh 1 hr	8	6.05E-11	4.57E-12	8	5.55E-11	7.13E-12	8	8.24E-12	1.14E-12	7	8.84E-12	1.45E-12	8	2.70E-11	3.97E-12	8	1.83E-11	3.29E-12
	Veh 3 hr	8	6.23E-11	4.69E-12	8	5.51E-11	6.49E-12	7	8.69E-12	1.03E-12	8	8.57E-12	1.01E-12	8	1.82E-11	1.60E-12	8	2.80E-11	3.37E-12
	CG 1 hr	6	5.79E-11	4.38E-12	8	6.83E-11	5.08E-12	8	9.01E-12	1.06E-12	7	1.11E-11	1.23E-12	8	2.09E-11	2.36E-12	8	2.71E-11	5.09E-12
	CG 3 hr	8	6.69E-11	6.85E-12	7	5.97E-11	5.76E-12	8	8.21E-12	7.64E-13	8	9.20E-12	1.29E-12	8	1.83E-11	1.30E-12	7	3.23E-11	5.18E-12
N-docosahexaenoyl serine	Veh 1 hr	7	7.03E-11	2.78E-12	8	8.87E-11	1.41E-11	8	1.48E-11	1.91E-12	7	2.06E-11	3.14E-12	8	4.72E-11	5.30E-12	8	1.66E-10	1.91E-11
	Veh 3 hr	8	8.86E-11	9.04E-12	8	7.32E-11	9.62E-12	8	1.45E-11	1.25E-12	8	2.01E-11	2.51E-12	8	3.88E-11	5.11E-12	8	2.26E-10	1.74E-11
	CG 1 hr	6	7.69E-11	7.90E-12	8	8.49E-11	1.56E-11	8	1.64E-11	1.81E-12	8	2.20E-11	2.42E-12	8	4.40E-11	5.41E-12	8	2.27E-10	2.05E-11
	CG 3 hr	8	9.29E-11	8.24E-12	7	8.25E-11	1.87E-11	8	1.65E-11	1.51E-12	8	1.92E-11	2.48E-12	8	4.10E-11	4.21E-12	7	2.26E-10	2.33E-11
***N*-acyl threonine**																			
N-palmitoyl threonine	Veh 1 hr	8	1.81E-10	5.37E-12	8	1.84E-10	2.52E-11		N/A			N/A			N/A			N/A	
	Veh 3 hr	8	1.81E-10	1.37E-11	8	1.46E-10	2.20E-11		N/A			N/A			N/A			N/A	
	CG 1 hr	7	1.65E-10	5.64E-12	8	1.24E-10	2.04E-11		N/A			N/A			N/A			N/A	
	CG 3 hr	7	2.05E-10	4.64E-12	7	1.60E-10	3.69E-11		N/A			N/A			N/A			N/A	
N-oleoyl threonine	Veh 1 hr	8	5.61E-11	9.89E-12	8	PISSR			N/A			N/A			N/A			N/A	
	Veh 3 hr	7	4.90E-11	3.81E-12	8	PISSR			N/A			N/A			N/A			N/A	
	CG 1 hr	5	4.00E-11	9.71E-13	8	PISSR			N/A			N/A			N/A			N/A	
	CG 3 hr	8	5.35E-11	3.47E-12	8	PISSR			N/A			N/A			N/A			N/A	
***N*-acyl tryptophan**																			
N-palmitoyl tryptophan	Veh 1 hr	8	3.68E-11	2.86E-12	7	2.09E-11	1.36E-12	8	8.28E-12	1.52E-12	8	1.22E-11	2.22E-12	8	6.12E-11	3.37E-12	8	1.03E-11	2.04E-12
	Veh 3 hr	8	4.44E-11	4.39E-12	7	2.75E-11	2.02E-12	8	8.94E-12	1.87E-12	8	1.67E-11	2.77E-12	8	6.16E-11	3.32E-12	8	1.65E-11	3.25E-12
	CG 1 hr	7	3.85E-11	2.37E-12	7	2.64E-11	1.22E-12	8	8.69E-12	2.12E-12	8	1.93E-11	2.86E-12	8	6.15E-11	5.35E-12	8	1.53E-11	2.92E-12
	CG 3 hr	8	4.36E-11	2.87E-12	6	2.40E-11	2.26E-12	8	9.04E-12	1.55E-12	8	1.63E-11	2.80E-12	8	6.52E-11	6.82E-12	7	1.58E-11	2.59E-12
N-stearoyl tryptophan	Veh 1 hr	8	8.46E-12	7.62E-13	8	3.00E-12	3.32E-13	8	4.00E-12	2.66E-13	8	2.40E-12	2.56E-13	8	4.90E-12	9.79E-13	8	2.53E-12	3.67E-13
	Veh 3 hr	8	8.05E-12	6.71E-13	8	2.78E-12	3.43E-13	8	4.22E-12	2.82E-13	8	2.90E-12	2.37E-13	8	5.95E-12	6.09E-13	8	2.80E-12	3.58E-13
	CG 1 hr	7	7.10E-12	7.03E-13	8	3.09E-12	2.80E-13	8	4.09E-12	2.51E-13	8	3.41E-12	1.90E-13	8	6.05E-12	8.72E-13	8	2.74E-12	3.58E-13
	CG 3 hr	8	7.62E-12	6.47E-13	7	2.53E-12	3.55E-13	8	5.70E-12	4.79E-13	8	2.99E-12	3.61E-13	8	7.21E-12	4.39E-13	7	3.21E-12	3.09E-13
N-oleoyl tryptophan	Veh 1 hr	8	1.94E-12	5.25E-13	8	1.29E-12	2.28E-13	8	1.42E-12	1.83E-13	8	1.41E-12	2.78E-13	8	7.66E-13	8.98E-14	8	1.65E-12	2.55E-13
	Veh 3 hr	8	2.63E-12	4.43E-13	7	9.40E-13	8.05E-14	8	1.76E-12	2.51E-13	8	1.97E-12	2.09E-13	8	9.36E-13	1.22E-13	8	2.31E-12	4.03E-13
	CG 1 hr	7	2.27E-12	3.33E-13	8	1.76E-12	3.18E-13	8	1.44E-12	2.02E-13	8	2.53E-12	2.09E-13	8	8.55E-13	8.32E-14	8	1.87E-12	2.27E-13
	CG 3 hr	8	2.39E-12	3.13E-13	6	1.27E-12	1.46E-13	8	1.91E-12	2.23E-13	8	1.99E-12	3.33E-13	8	7.34E-13	1.13E-13	7	2.36E-12	3.17E-13
N-linoleoyl tryptophan	Veh 1 hr	8	1.75E-12	2.09E-13	8	BDL			N/A			N/A			N/A			N/A	
	Veh 3 hr	8	1.71E-12	2.24E-13	8	BDL			N/A			N/A			N/A			N/A	
	CG 1 hr	7	1.65E-12	2.49E-13	8	BDL			N/A			N/A			N/A			N/A	
	CG 3 hr	8	1.98E-12	1.16E-13	8	BDL			N/A			N/A			N/A			N/A	
N-arachidonoyl tryptophan	Veh 1 hr	8	BDL		8	BDL		8	BDL		8	BDL		8	1.24E-11	8.18E-13	8	BDL	
	Veh 3 hr	8	BDL		8	BDL		8	BDL		8	BDL		8	1.08E-11	7.89E-13	8	BDL	
	CG 1 hr	8	BDL		8	BDL		8	BDL		8	BDL		8	1.14E-11	5.16E-13	8	BDL	
	CG 3 hr	8	BDL		8	BDL		8	BDL		8	BDL		8	1.26E-11	1.27E-12	7	BDL	

N-docosahexaenoyl tryptophan	Veh 1 hr	8	BDL		8	BDL			N/A			N/A			N/A			N/A	
	Veh 3 hr	8	BDL		8	BDL			N/A			N/A			N/A			N/A	
	CG 1 hr	8	BDL		8	BDL			N/A			N/A			N/A			N/A	
	CG 3 hr	8	BDL		8	BDL			N/A			N/A			N/A			N/A	
***N*-acyl tyrosine**																			
N-palmitoyl tyrosine	Veh 1 hr	8	2.95E-12	5.15E-13	8	4.30E-12	4.48E-13	8	3.74E-12	2.69E-13	8	2.83E-12	2.74E-13	8	6.08E-12	7.34E-13	8	7.70E-13	1.54E-13
	Veh 3 hr	8	3.26E-12	3.89E-13	8	5.02E-12	3.12E-13	8	3.84E-12	3.21E-13	8	2.94E-12	2.51E-13	8	5.45E-12	5.41E-13	8	7.20E-13	1.22E-13
	CG 1 hr	7	2.83E-12	3.93E-13	8	3.71E-12	4.07E-13	8	3.77E-12	3.77E-13	8	3.16E-12	1.79E-13	8	6.10E-12	5.49E-13	8	4.50E-13	6.31E-14
	CG 3 hr	8	3.20E-12	6.48E-13	6	4.89E-12	3.21E-13	8	4.25E-12	3.20E-13	8	3.03E-12	2.93E-13	8	6.59E-12	7.04E-13	7	8.52E-13	1.64E-13
N-stearoyl tyrosine	Veh 1 hr	8	BDL		8	2.03E-12	3.04E-13	8	2.63E-12	2.88E-13	8	2.11E-12	4.43E-13	8	4.29E-12	9.00E-13	8	1.04E-12	2.59E-13
	Veh 3 hr	8	BDL		8	2.23E-12	2.37E-13	8	2.88E-12	2.71E-13	8	1.78E-12	3.16E-13	8	3.39E-12	7.16E-13	8	1.28E-12	2.68E-13
	CG 1 hr	8	BDL		8	1.80E-12	2.63E-13	8	2.74E-12	2.47E-13	8	1.88E-12	4.02E-13	8	3.76E-12	9.28E-13	8	1.41E-12	2.79E-13
	CG 3 hr	8	BDL		7	1.78E-12	3.34E-13	8	3.50E-12	5.25E-13	8	1.67E-12	2.47E-13	8	3.89E-12	8.48E-13	7	1.30E-12	2.56E-13
N-oleoyl tyrosine	Veh 1 hr	8	PISSR		8	PISSR		8	2.14E-12	1.06E-13	8	2.04E-12	1.78E-13	8	4.34E-12	4.29E-13	8	3.58E-13	1.29E-13
	Veh 3 hr	8	PISSR		8	PISSR		8	2.09E-12	1.25E-13	8	2.05E-12	3.37E-13	8	3.78E-12	6.43E-13	8	2.32E-13	5.86E-14
	CG 1 hr	8	PISSR		8	PISSR		8	2.19E-12	1.78E-13	8	2.15E-12	2.67E-13	8	4.24E-12	6.61E-13	8	3.60E-13	7.65E-14
	CG 3 hr	8	PISSR		8	PISSR		8	2.58E-12	2.00E-13	8	1.90E-12	2.83E-13	8	4.17E-12	7.01E-13	7	2.70E-13	1.02E-13
N-linoleoyl tyrosine	Veh 1 hr	8	BDL		8	BDL		8	PISSR		8	PISSR		8	PISSR		8	PISSR	
	Veh 3 hr	8	BDL		8	BDL		8	PISSR		8	PISSR		8	PISSR		8	PISSR	
	CG 1 hr	8	BDL		8	BDL		8	PISSR		8	PISSR		8	PISSR		8	PISSR	
	CG 3 hr	8	BDL		8	BDL		8	PISSR		8	PISSR		8	PISSR		7	PISSR	
N-arachidonoyl tyrosine	Veh 1 hr	8	7.85E-13	7.68E-14	8	PISSR		8	6.70E-13	8.48E-14	8	4.41E-13	7.17E-14	8	9.72E-13	1.87E-13	8	1.05E-12	1.52E-13
	Veh 3 hr	8	7.56E-13	1.34E-13	8	PISSR		8	6.79E-13	8.08E-14	8	4.94E-13	8.18E-14	8	9.09E-13	1.60E-13	8	1.07E-12	1.44E-13
	CG 1 hr	7	7.68E-13	7.40E-14	8	PISSR		8	5.47E-13	9.58E-14	8	6.73E-13	8.43E-14	8	1.28E-12	1.65E-13	8	8.25E-13	1.05E-13
	CG 3 hr	8	6.29E-13	9.78E-14	8	PISSR		8	4.49E-13	7.56E-14	8	4.48E-13	4.54E-14	8	9.78E-13	1.11E-13	7	8.71E-13	1.07E-13
N-docosahexaenoyl tyrosine	Veh 1 hr		N/A			N/A		8	BDL		8	BDL		8	BDL		7	BDL	
	Veh 3 hr		N/A			N/A		8	BDL		8	BDL		8	BDL		8	BDL	
	CG 1 hr		N/A			N/A		8	BDL		8	BDL		8	BDL		8	BDL	
	CG 3 hr		N/A			N/A		8	BDL		8	BDL		8	BDL		7	BDL	
***N*-acyl valine**																			
N-palmitoyl valine	Veh 1 hr	7	3.05E-11	1.44E-12	8	2.22E-11	1.15E-12	8	5.48E-12	9.52E-13	8	7.86E-12	8.88E-13	8	1.57E-11	1.06E-12	8	1.21E-11	3.07E-12
	Veh 3 hr	8	2.97E-11	1.43E-12	8	2.16E-11	2.45E-12	8	4.91E-12	8.42E-13	8	7.39E-12	6.47E-13	8	1.38E-11	1.45E-12	8	1.21E-11	1.43E-12
	CG 1 hr	7	3.33E-11	1.98E-12	8	2.09E-11	1.96E-12	8	5.04E-12	9.57E-13	8	8.05E-12	5.98E-13	8	1.44E-11	7.11E-13	8	1.37E-11	2.71E-12
	CG 3 hr	8	3.64E-11	2.67E-12	7	2.53E-11	1.62E-12	8	6.11E-12	1.10E-12	8	7.16E-12	7.55E-13	8	1.46E-11	1.71E-12	7	1.60E-11	5.02E-12
N- stearoyl valine	Veh 1 hr	8	1.47E-11	1.36E-12	8	1.13E-11	4.84E-13	8	3.30E-13	5.01E-14	8	5.58E-12	5.49E-13	8	1.01E-11	6.35E-13	8	1.12E-11	2.35E-12
	Veh 3 hr	8	1.30E-11	7.56E-13	8	1.08E-11	9.58E-13	7	2.49E-13	1.02E-13	8	4.67E-12	3.31E-13	8	8.06E-12	3.18E-13	8	1.02E-11	1.57E-12
	CG 1 hr	7	1.37E-11	9.04E-13	7	9.34E-12	4.15E-13	7	4.03E-13	1.07E-13	8	5.40E-12	3.71E-13	8	8.62E-12	6.71E-13	8	1.07E-11	1.90E-12
	CG 3 hr	8	1.52E-11	1.95E-12	6	1.17E-11	5.20E-13	8	2.99E-13	6.62E-14	8	5.13E-12	7.02E-13	8	9.08E-12	6.63E-13	7	1.34E-11	3.38E-12
N-oleoyl valine	Veh 1 hr		N/A		8	8.38E-11	7.91E-12	7	6.14E-06	6.13E-06	8	1.04E-03	5.32E-04	8	4.94E-05	2.99E-05	8	5.51E-10	2.21E-10
	Veh 3 hr		N/A		8	9.31E-11	8.61E-12	7	6.08E-07	6.08E-07	8	2.09E-04	9.69E-05	8	2.49E-06	8.81E-07	8	3.34E-10	4.17E-11

	CG 1 hr		N/A		8	7.46E-11	6.28E-12	7	2.46E-06	2.46E-06	8	1.38E-04	4.32E-05	8	4.74E-05	2.04E-05	8	2.53E-10	2.37E-11
	CG 3 hr		N/A		7	8.66E-11	6.34E-12	7	7.24E-08	6.39E-08	8	4.33E-03	4.23E-03	8	7.58E-05	4.51E-05	7	4.19E-10	9.07E-11
N-nervonoyl valine	Veh 1 hr	8	BDL		8	BDL		8	BDL		8	BDL		8	BDL		8	BDL	
	Veh 3 hr	8	BDL		8	BDL		8	BDL		8	BDL		8	BDL		8	BDL	
	CG 1 hr	8	BDL		8	BDL		8	BDL		8	BDL		8	BDL		8	BDL	
	CG 3 hr	8	BDL		8	BDL		8	BDL		8	BDL		8	BDL		7	BDL	
N-linoleoyl valine	Veh 1 hr	8	BDL		8	BDL		8	PISSR		7	1.44E-13	2.95E-14	8	PISSR		8	PISSR	
	Veh 3 hr	8	BDL		8	BDL		8	PISSR		7	1.43E-13	2.77E-14	8	PISSR		8	PISSR	
	CG 1 hr	8	BDL		8	BDL		8	PISSR		7	1.70E-13	2.36E-14	8	PISSR		8	PISSR	
	CG 3 hr	8	BDL		8	BDL		8	PISSR		7	1.88E-13	2.76E-14	8	PISSR		7	PISSR	
N-docosahexaenoyl valine	Veh 1 hr	8	BDL		8	BDL		8	5.46E-13	1.29E-13	7	4.96E-13	7.72E-14	8	PISSR		8	PISSR	
	Veh 3 hr	8	BDL		8	BDL		6	4.20E-13	1.12E-13	8	3.51E-13	7.55E-14	8	PISSR		8	PISSR	
	CG 1 hr	8	BDL		8	BDL		8	5.99E-13	1.49E-13	8	4.49E-13	5.13E-14	8	PISSR		8	PISSR	
	CG 3 hr	8	BDL		8	BDL		8	9.09E-13	1.31E-13	8	4.74E-13	9.70E-14	8	PISSR		7	PISSR	

Supplemental Figure 1

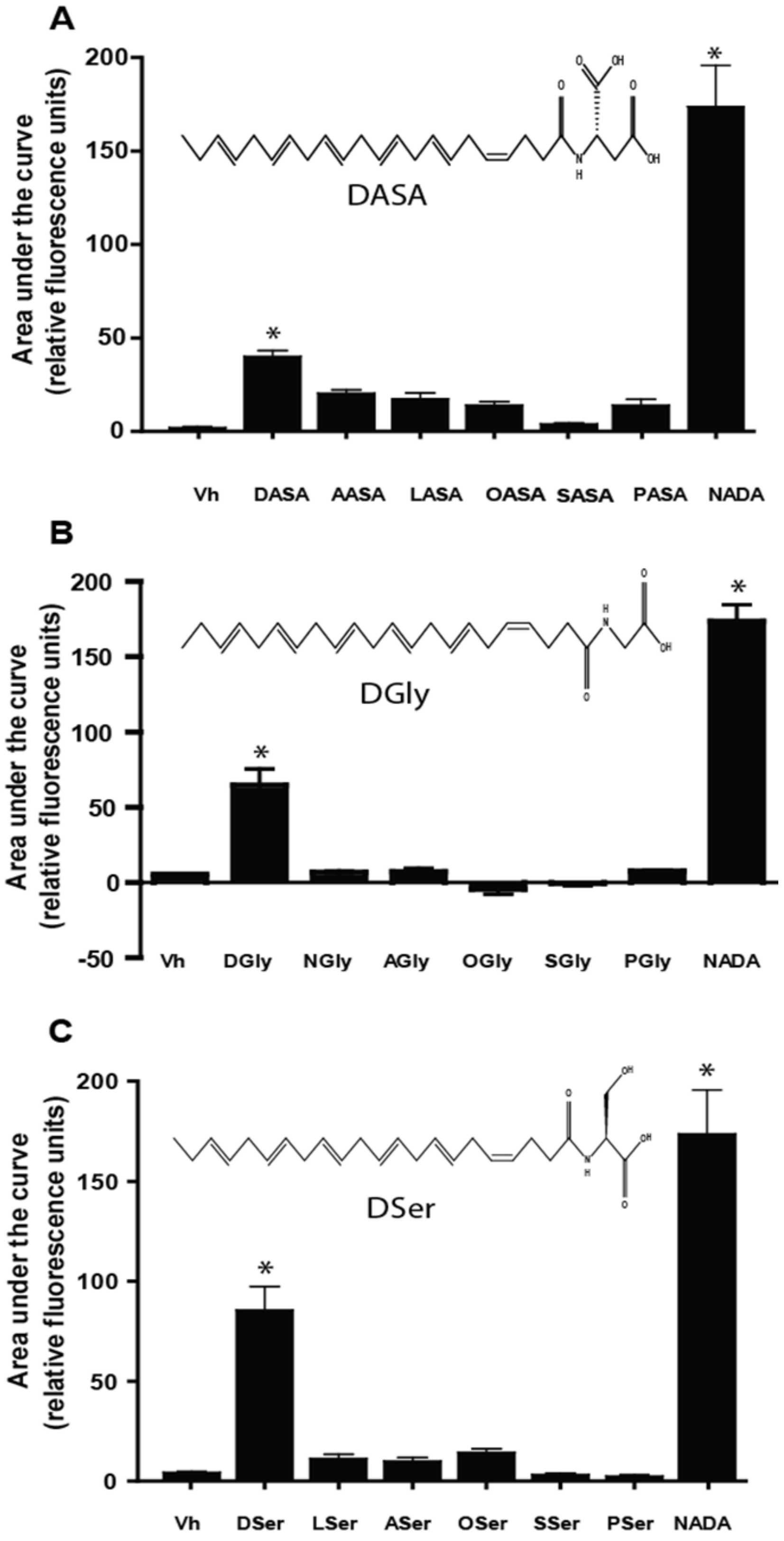

Supplemental Figure 2

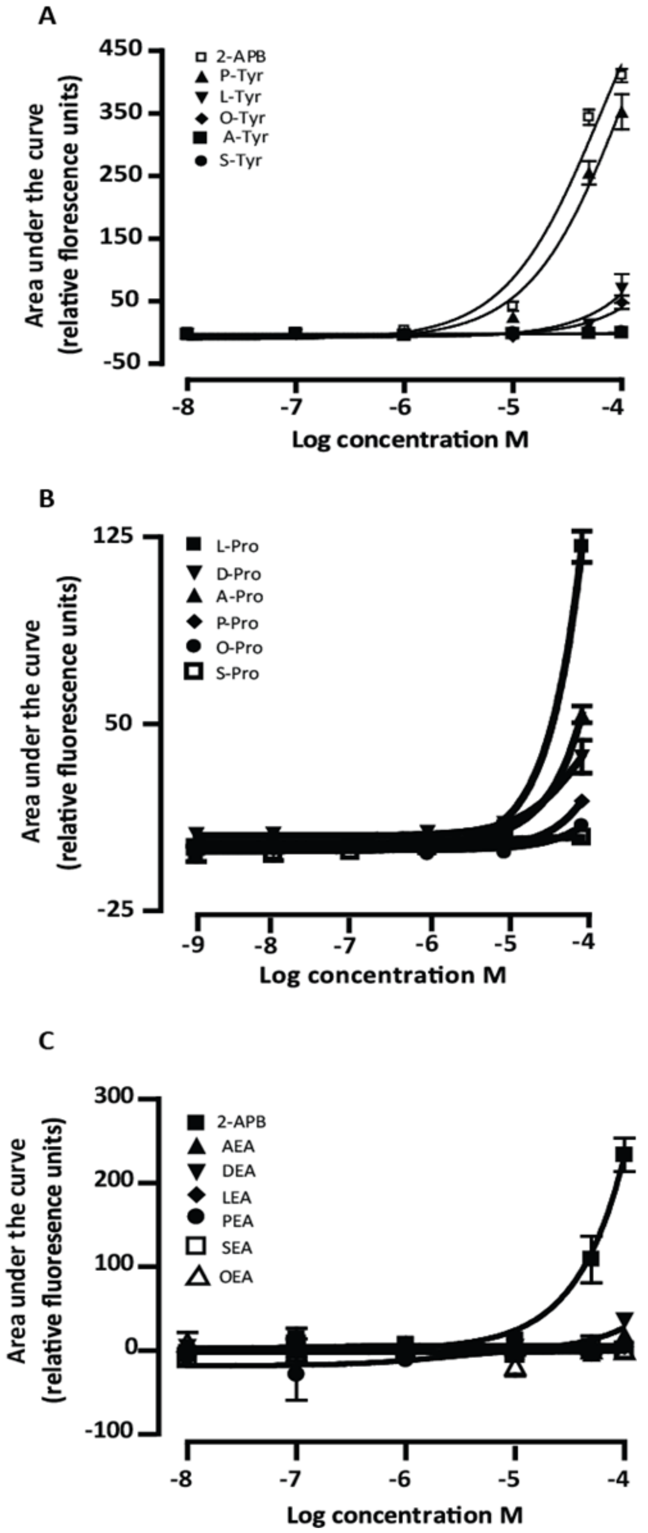

Supplemental Figure 3

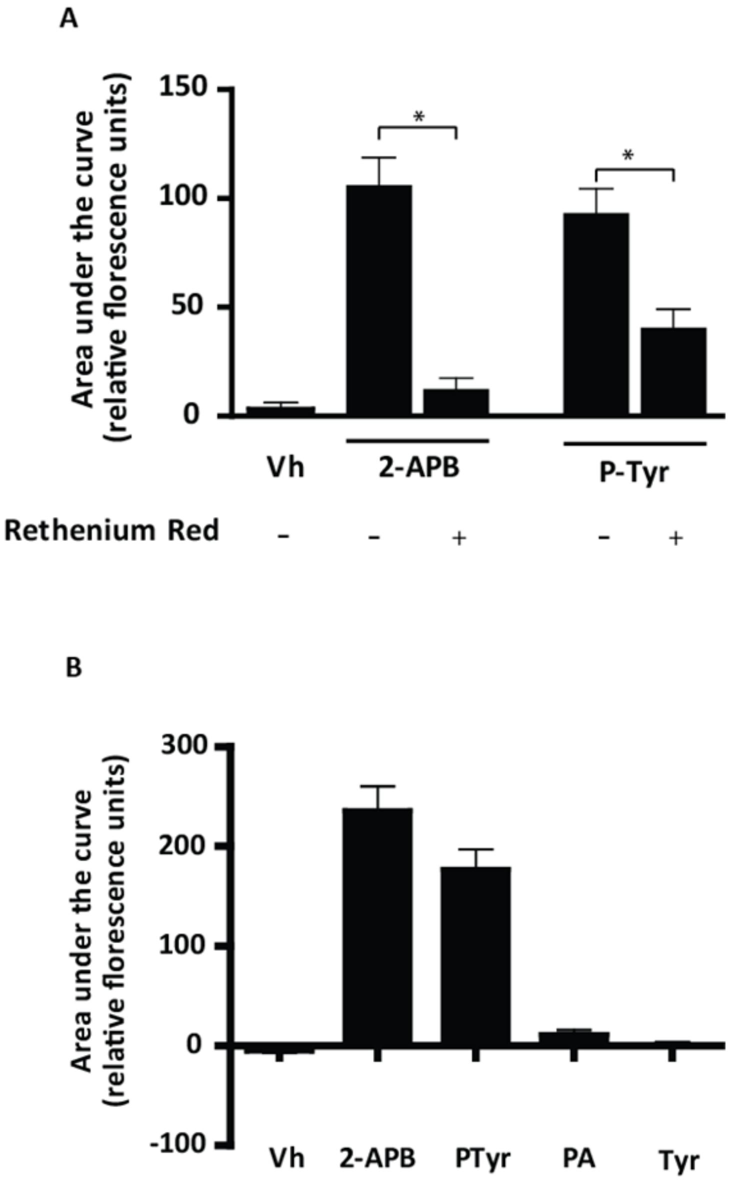

Supplemental Figure 4

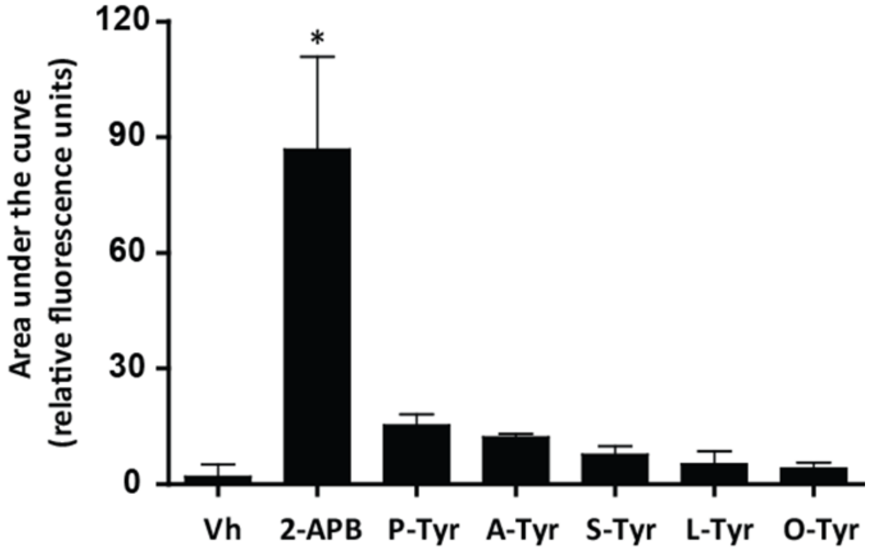

Supplemental Figure 5

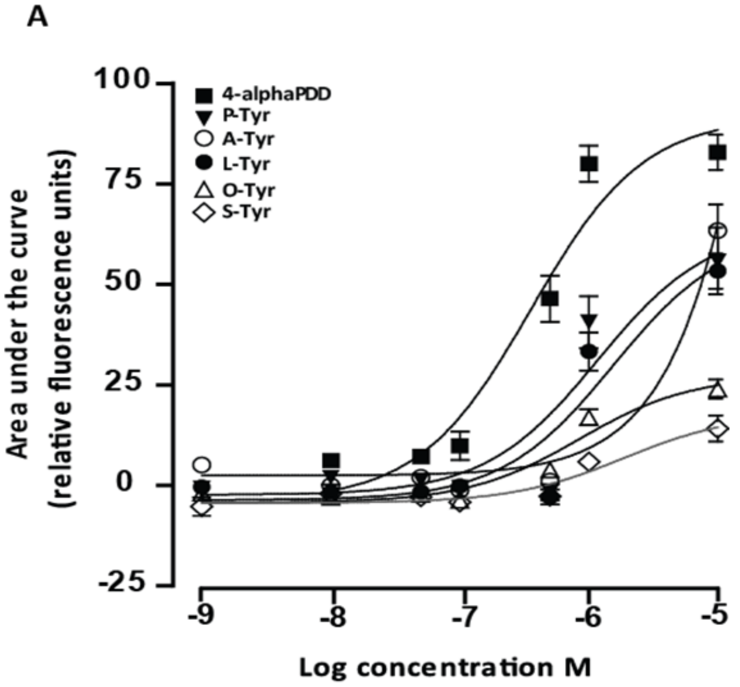

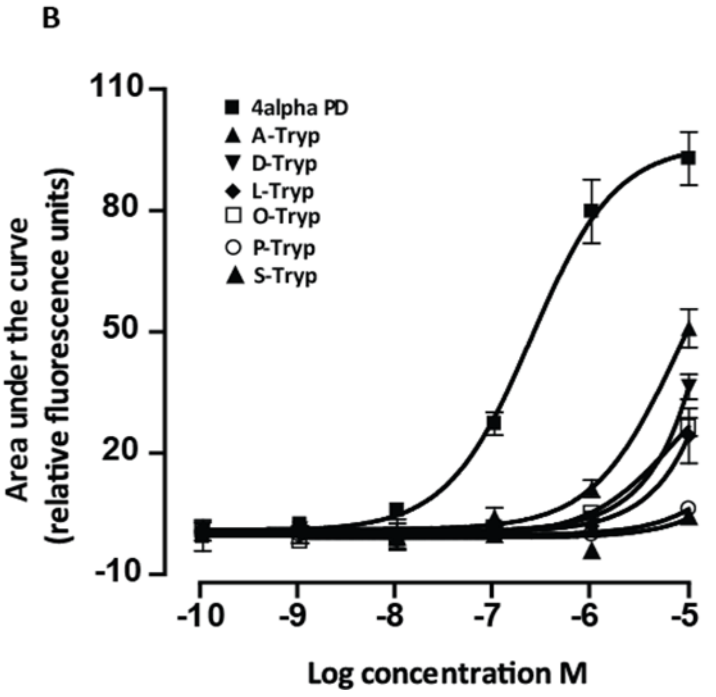

Supplemental Figure 6

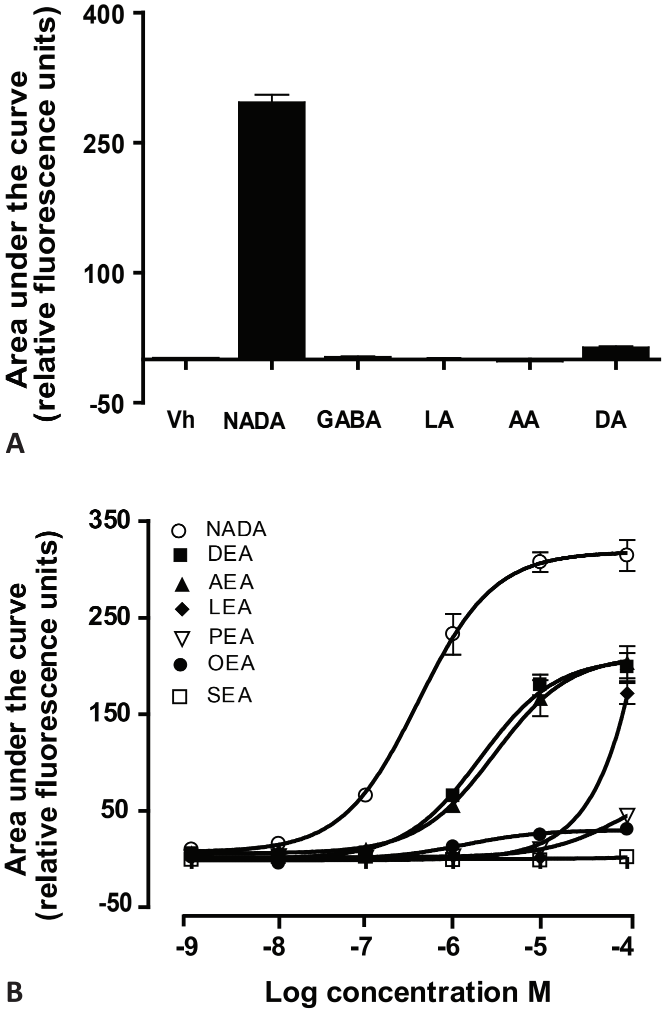

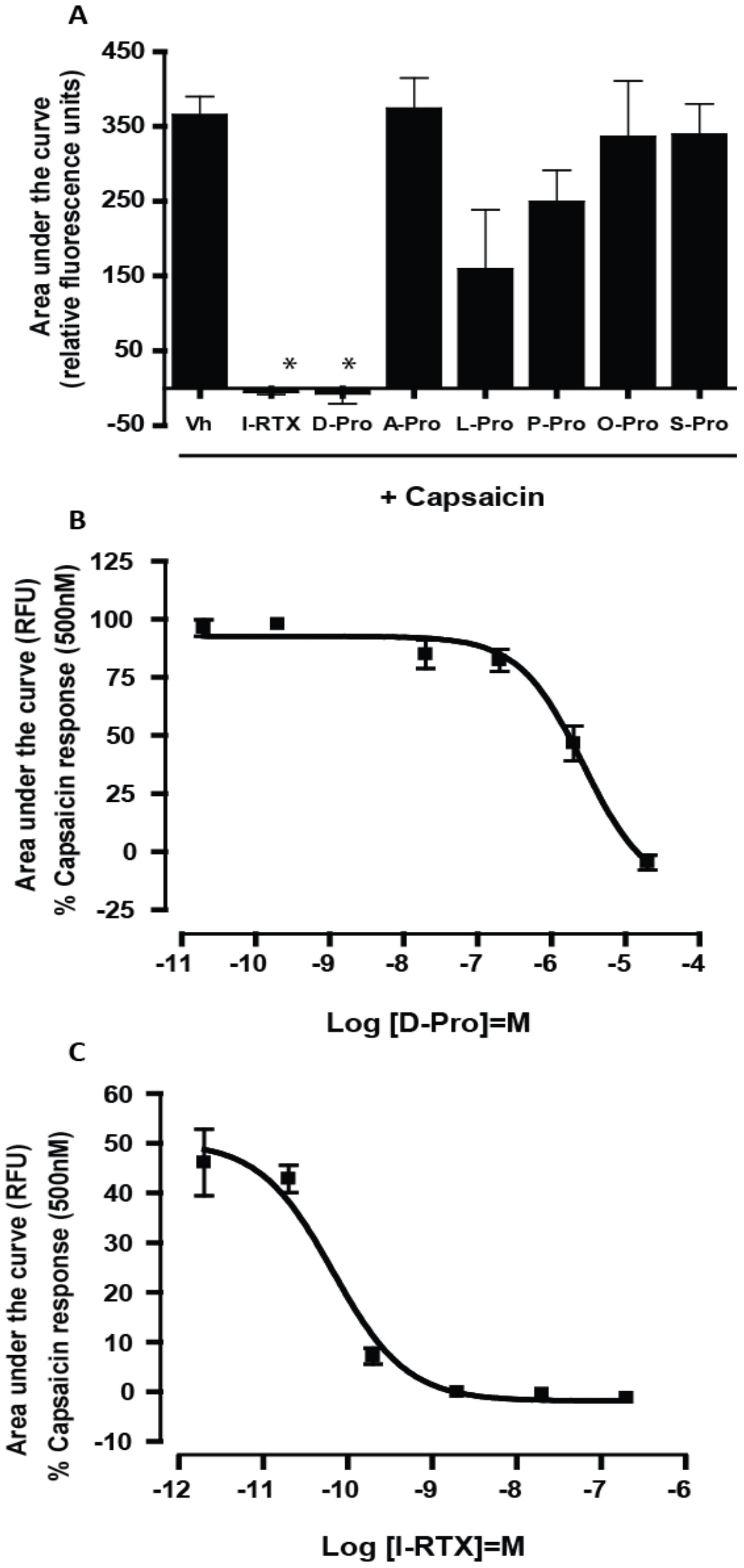

Supplemental Figure 7

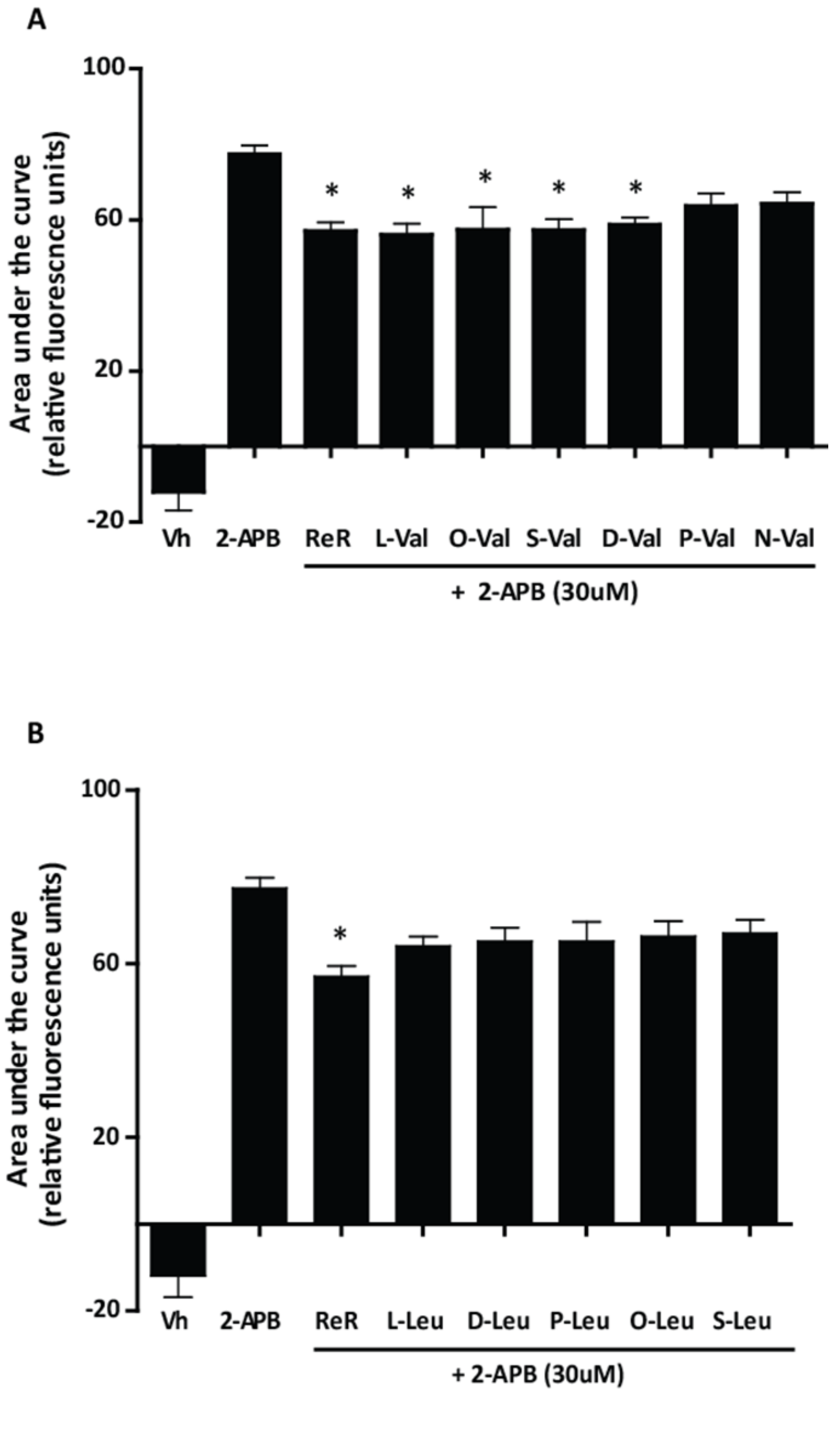

Supplemental Figure 8

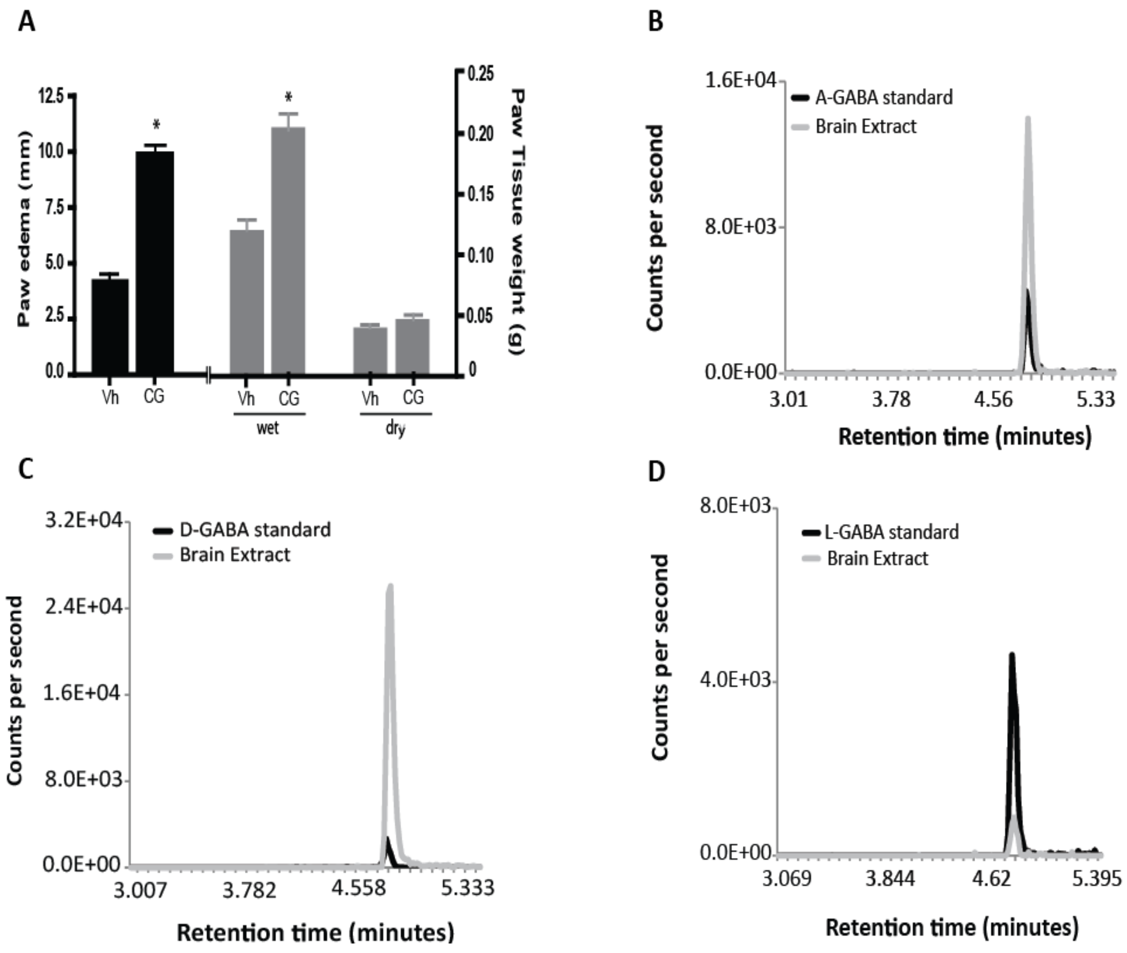

Supplemental Figure 9

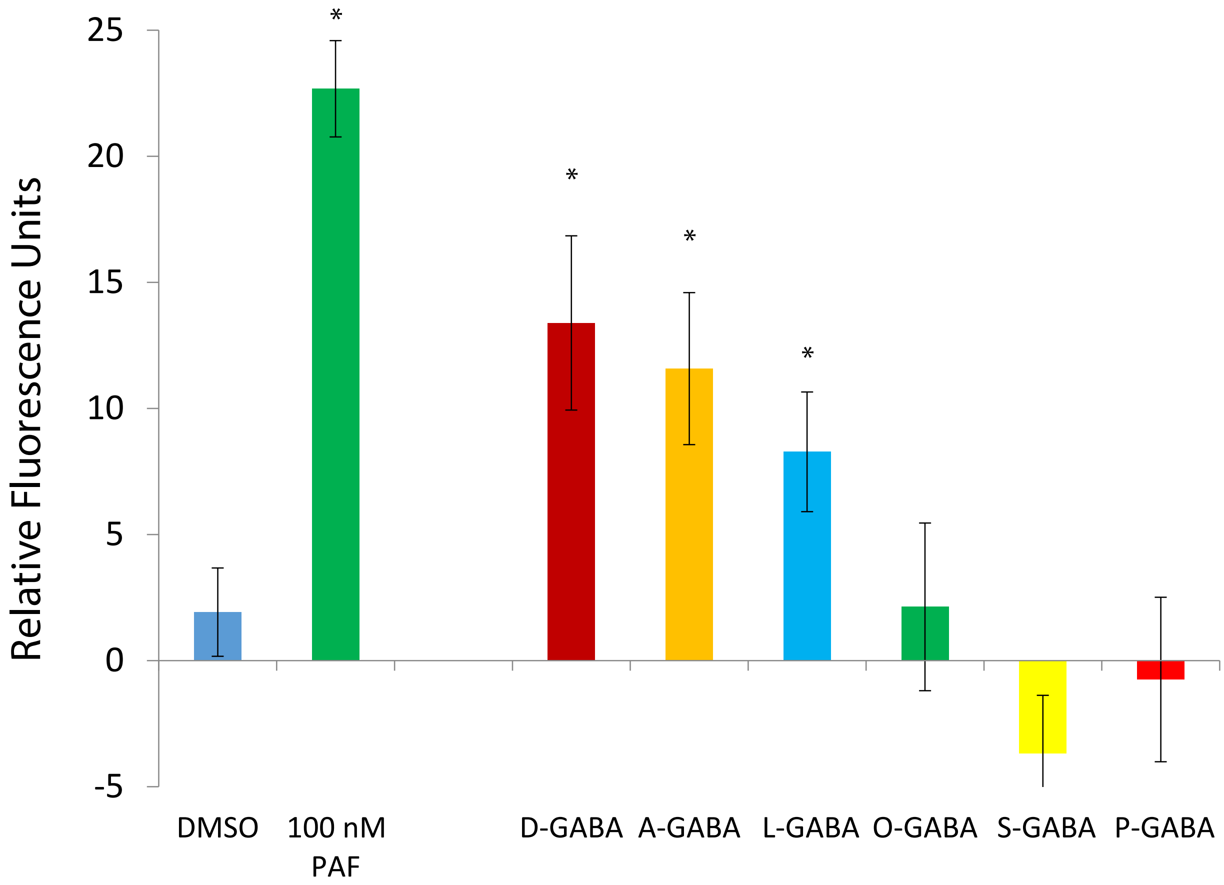

Supplemental Figure 10

Supplemental Figure Legends

Supplemental Table 1. List of *N*-acyl amides used in calcium imaging screens. Each of the *N*-acyl amides used in calcium imaging screens was grouped into families categorized by their amine as listed here. This is not an exhaustive list of all possible *N*-acyl amides, but rather a list of those that have been made in-house here for these studies.

Supplemental Table 2: Mean levels of each lipid in the screening library, if detected, for each treatment group, including the N and standard error for each group. The treatment groups were vehicle 1 hour (Veh 1 hr), vehicle 3 hours (Veh 3 hr), carrageenan 1 hour (CG 1 hr), and carrageenan 3 hours (CG 3 hr). The group means are in moles of analyte per gram of tissue. Some of the mean values for the carrageenan groups (1 and 3 hours) are shaded either green or red: If a mean value is shaded green, then there was a significant increase ($p<.05$) in the corresponding lipid's levels compared to vehicle injection at the same time point and if a value is shaded red, then there was a significant decrease in that lipid's levels. If there is no mean value listed, then the lipid was either present in some samples at random (PISSR), below detectable levels (BDL), or simply was not run or was not applicable to the brain region (N/A).

Supplemental Figure 1: Level of calcium mobilization in TRPV1-transfected HEK293 cells by 10µM of individual members of *N*-acyl aspartic acid and *N*-acyl serine families of lipids. **(A)** *N*-acyl aspartic acid: *N*-docosahexaenoyl aspartic acid (DASA), *N*-arachidonoyl aspartic acid (AASA), *N*-linoleoyl aspartic acid (LASA), *N*-oleoyl aspartic acid (OASA), *N*-palmitoyl aspartic acid (PASA) and *N*-stearoyl aspartic acid (SASA). **(B)** *N*-acyl glycine: *N*-docosahexaenoyl glycine (DGly), *N*-arachidonoyl glycine (AGly), *N*-nervonoyl glycine (NGLY), *N*-oleoyl glycine (OGly), *N*-palmitoyl glycine (PGly) and *N*-stearoyl glycine (SGly). **(C)** *N*-acyl serines: *N*-docosahexaenoyl serine (DSer), *N*-arachidonoyl serine (ASer), *N*-linoleoyl serine (LSer), *N*-oleoyl serine (OSer), *N*-stearoyl seine (SSer) and *N*-palmitoyl serine (PSer).Data were calculated as area under the curve of relative fluorescence units and are presented as mean +/- standard error of the mean (SEM) averaged over three different experiments. * $p<0.05$ from control.

Supplemental Figure 2: Level of calcium mobilization in TRPV2-transfected HEK293 cells by individual members of *N*-acyl tyrosine, *N*-acyl proline and *N*-acyl ethanolamine**.** **(A)** *N*-acyl tyrosine including: *N*-palmitoyl tyrosine (P-Tyr), *N*-linoleoyl tyrosine (L-Tyr), *N*-arachidonoyl tyrosine (A-Tyr), *N*-oleoyl tyrosine (O-Tyr) and *N*-stearoyl tyrosine (S-Tyr). **(B)** *N*-acyl proline including: *N*-docosahexaenoyl proline (D-Pro), *N*-linoleoyl proline (L-Pro), *N*-arachidonoyl proline (A-Pro), *N*-palmitoyl proline (P-Pro), *N*-Oleoyl Proline (O-Pro) and *N*-Stearoyl Proline (S-Pro). **(C)** *N*-acyl ethanolamine including: *N*-arachidonoyl ethanolamine (AEA), *N*-docosahexaenoyl ethanolamine (DEA), *N*-linoleoyl ethanolamine (LEA), *N*-palmitoyl ethanolamine (PEA), *N*-stearoyl ethanolamine (SEA) and N-palmitoyl ethanolamine (PEA).

Supplemental Figure 3: *N*-acyl tyrosine responses at TRPV2. **(A)** Responses of 30 µM 2-APB or *N*-palmitoyl tyrosine (P-Tyr) in the presence and absence of 30 µM Rethenium Red (ReR). **(B)** Levels of calcium mobilization in response to 30 µM of 2-APB, *N*-palmitoyl tyrosine (P-Tyr), palmitic acid (PA), tyrosine (Tyr) or DMSO vehicle (Vh).

Supplemental Figure 4: Level of calcium mobilization in TRPV3-transfected HEK293 cells by individual members of *N*-acyl tyrosine including: *N*-palmitoyl tyrosine (P-Tyr), *N*-linoleoyl

tyrosine (L-Tyr), *N*-arachidonoyl tyrosine (A-Tyr), *N*-oleoyl tyrosine (O-Tyr) and *N*-stearoyl tyrosine (S-Tyr).

Supplemental Figure 5: Level of calcium mobilization in TRPV4-transfected HEK293 cells by individual members of *N*-acyl tyrosine and *N*-acyl tryptophan**. (A)** *N*-acyl tyrosine including: *N*-palmitoyl tyrosine (P-Tyr), *N*-linoleoyl tyrosine (L-Tyr), *N*-arachidonoyl tyrosine (A-Tyr), *N*-oleoyl tyrosine (O-Tyr) and *N*-stearoyl tyrosine (S-Tyr).**(B)** *N*-acyl tryptophan including: *N*-arachidonoyl tryptophan (A-Tryp), *N*-docosahexaenoyl tryptophan (D-Tryp), *N*-linoleoyl tryptophan (L-Tryp), *N*-oleoyl tryptophan (O-Tryp), *N*-palmitoyl tryptophan (P-Tryp) and *N*-stearoyl tryptophan (S-Tryp).

Supplemental Figure 6: Level of calcium mobilization in TRPV1-transfected HEK cells. (A) Comparison of agonist activity of vehicle and 10µM *N*-arachidonoyl dopamine (NADA), GABA, linoleic acid (LA), arachidonic acid (AA), and docosahexaenoic acid (DA) in TRPV1-HEK cells. (B) Concentration curve of calcium mobilization via Fura2AM in TRPV1-HEK cells using NADA, *N*-docosahexaenoyl ethanolamine (DEA), *N*-arachidonoyl ethanolamine (AEA), *N*-linoleoyl ethanolamine (LEA), *N*-oleoyl ethanolamine (OEA), *N*-palmitoyl ethanolamine (PEA) and *N*-stearoyl ethanolamine (SEA).

Supplemental Figure 7: Antagonist activity of individual *N*-acyl proline lipids at TRPV1. (A) Comparison of antagonist activity of the potent TRPV1 antagonist, I-RTX with individual members of *N*-acyl proline: *N*-docosahexaenoyl proline (D-Pro), *N*-arachidonoyl proline (A- Pro), *N*-linoleoyl proline (L- Pro), *N*-oleoyl proline (O- Pro), *N*-palmitoyl proline (P- Pro) and *N*-stearoyl proline (S- Pro) all in the presence of capsaicin. **(B)** Concentration curve of IRTX potency in the presence of Capsaicin. **(C)** Concentration curve of D-Pro in the presence of Capsaicin.

Supplemental Figure 8: (A) Level of calcium mobilization by 30 µM 2-APB in TRPV3-transfected HEK293 cells following 20 minute incubation with 10 µM individual *N*-acyl valines including: *N*-linoleoyl valine (L-Val), *N*-oleoyl valine (O-Val), *N*-stearoyl valine (S-Val), *N*-docosahexaenoyl valine (D-Val), *N*-palmitoyl valine (P-Val) and *N*-nervonoyl valine (N-Val). **(B)** *N*-acyl leucine including: *N*-linoleoyl leucine (L-Leu), *N*-docosahexaenoyl leucine (D-Leu), *N*-palmitoyl leucine (P-Leu), *N*-oleoyl leucine (O-Leu) and N-stearoyl leucine (S-Leu). Data are presented as mean +/- standard error of the mean (SEM) averaged over three different experiments. *$p<0.05$

Supplemental Figure 9: Effects of carrageenan injection on paw edema and brain lipids. (A) Levels of paw edema on injected side (vehicle: VH and carrageenan: CG). The paws were also analyzed for their wet and dry weight as a measure of how these values relate to the treatment. (B-D) Overlay chromatograms of *N*-arachidonoyl GABA (A-GABA), *N*-docosahexaenoyl GABA (D-GABA), and *N*-linoleoyl GABA in cerebellar brain tissue compared to standards. Lines in bold black are those of the standards and those in grey are from extract injections.

Supplemental Figure 10: Level of calcium mobilization on BV-2 microglia with individual *N*-acyl GABA lipids. Calcium mobilization in BV2 cells as measured through the change in relative fluorescence units using Fura2AM. Platelet activating factor (PAF) was used as a positive control and compared to 10µM *N*-docosahexaenoyl GABA (D-GABA), *N*-arachidonoyl GABA (A GABA), *N*-linoleoyl GABA (L GABA), *N*-oleoyl GABA (O GABA), *N*-palmitoyl GABA (P GABA) and N-stearoyl GABA (S GABA). *$p<0.05$

Supplemental Figure Legends
